# Impact of Lytic Phages on Phosphorus- vs. Nitrogen-Limited Marine Microbes

**DOI:** 10.3389/fmicb.2020.00221

**Published:** 2020-02-21

**Authors:** Julie Pourtois, Corina E. Tarnita, Juan A. Bonachela

**Affiliations:** ^1^Department of Biology, Stanford University, Stanford, CA, United States; ^2^Department of Ecology and Evolutionary Biology, Princeton University, Princeton, NJ, United States; ^3^Department of Ecology, Evolution, and Natural Resources, Rutgers University, New Brunswick, NJ, United States

**Keywords:** marine phages, marine bacteria, nutrient limitation, virus-to-prokaryote ratio, carbon sink

## Abstract

Lytic viruses kill almost 20% of marine bacteria every day, re-routing nutrients away from the higher trophic levels of the marine food web and back in the microbial loop. Importantly, the effect of this inflow of key elements on the ecosystem depends on the nutrient requirements of bacteria as well as on the elemental composition of the viruses that infect them. Therefore, the influence of viruses on the ecosystem could vary depending on which nutrient is limiting. In this paper, we considered an existing multitrophic model (nutrient, bacteria, zooplankton, and viruses) that accounts for nitrogen limitation, and developed a phosphorus-limited version to assess whether the limiting nutrient alters the role of viruses in the ecosystem. For both versions, we evaluated the stationary state of the system with and without viruses. In agreement with existing results, nutrient release increased with viruses for nitrogen–limited systems, while zooplankton abundance and export to higher trophic levels decreased. We found this to be true also for phosphorus-limited systems, although nutrient release increased less than in nitrogen-limited systems. The latter supports a nutrient-specific response of the ecosystem to viruses. Bacterial concentration decreased in the phosphorus-limited system but increased in most nitrogen-limited cases due to a switch from mostly bottom-up to entirely top-down control by viruses. Our results also show that viral concentration is best predicted by a power-law of bacterial concentration with exponent different from 1. Finally, we found a positive correlation between carbon export and viruses regardless of the limiting nutrient, which led us to suggest viral abundance as a predictor of carbon sink.

## Introduction

Viruses are the most abundant biological entities on Earth. Although long-accepted that phages outnumber bacteria by a factor of 10 in marine ecosystems (Chibani-chennoufi et al., 2004), recent studies have shown that the virus-to-prokaryote ratio (VPR) can in fact vary over multiple orders of magnitude (Wigington et al., 2016; Parikka et al., 2017). Viruses exert top-down control of bacteria (Middelboe and Lyck, 2002), but their impact on the microbial community goes beyond the mortality of individual cells. When lysing their hosts, viruses cause the release of dissolved and particulate organic matter that would otherwise be exported from the system, a process known as the “viral shunt” (Fuhrman, 1999; Wilhelm and Suttle, 1999; Middelboe and Lyck, 2002; Suttle, 2007; Miki et al., 2008; Keller and Hood, 2011, 2013; Weitz and Wilhelm, 2012; Weitz et al., 2015). The dissolved organic matter (DOM) released by this viral shunt can be ultimately reused by uninfected bacteria and phytoplankton, resulting in an additional bottom-up source of regulation that affects the entire microbial community (Middelboe et al., 1996; Shelford et al., 2012; Weitz et al., 2015).

The top-down and bottom-up effects of viruses on the microbial community have ramifications for important ecosystem functions, such as productivity and the carbon sink. Productivity (i.e., the rate of biomass increase) is affected by both bacterial abundance, which is negatively impacted by viruses via mortality (Middelboe and Lyck, 2002), and by bacterial growth, which is positively impacted by viruses via the increase in nutrient concentrations (Middelboe et al., 1996). Although there is broad agreement on these two individual processes, there is a lack of consensus regarding the net effect of viruses on primary productivity. Recent empirical observations (Weinbauer et al., 2011; Shelford et al., 2012) and theoretical predictions (Weitz et al., 2015) suggest that viruses increase primary productivity, which is in conflict with previous findings (Suttle et al., 1990, Middelboe and Lyck, 2002). In addition, viruses' impact on the carbon sink is also 2-fold. On the one hand, lysis can diminish the contribution of zooplankton to the carbon sink (via fecal pellet production) by reducing the availability and therefore the consumption of bacteria by zooplankton (Fuhrman, 1992). These rapidly sinking pellets account for about half of the vertical carbon flux, but also contain other nutrients such as nitrogen and phosphorus that are essential for microbial growth (Small et al., 1983; Lee and Fisher, 1994). On the other hand, lysis contributes to the carbon sink through the production of carbon-rich debris from, for example, cell walls that are difficult to recycle (Gobler et al., 1997; Suttle, 2007). In contrast to fecal pellet production, viral lysis allows for a separation of carbon from other nutrients. For example, nitrogen and phosphorus contained in the cell are released in their dissolved form, and can be re-used by other bacteria that can, in turn, be lysed. In contrast, carbon constitutes a large proportion of particulate debris, which sink more easily (Suttle, 2007). As a result, viruses increase the efficiency of the carbon sink—defined as the ratio of carbon to the limiting resource exported—although, given the viral 2-fold effect described above, it is unknown whether this increase in efficiency goes along with an increase in total carbon transport.

All nutrients are not affected in the same way by the viral shunt, adding complexity to this picture. The nutrients released during lysis result from unused host resources and machinery and leftovers from the production of progeny viruses (virions). The composition of this released material is thus influenced by the elemental composition of the organisms involved. Relative to viruses, bacteria have a higher protein-to-DNA ratio and, therefore, a higher nitrogen-to-phosphorus (N:P) ratio (Jover et al., 2014). The consequential lower N:P ratio of the viral progeny leads to a cell debris from lysis that has a higher N:P ratio than the original cell composition. This larger amount of organic N than organic P relative to the lysed cell is thus released and made available to other bacteria for growth. The bacterial biomass that is created from these released nutrients depends on which of the two nutrients influences growth the most (or, assuming Liebig's law, which one is limiting). Specifically, nutrient and growth are linked through host nutrient requirement, uptake dynamics, and resulting elemental stoichiometry, which vary across species and environmental conditions (Morel, 1987; Sterner and Elser, 2002; Bonachela et al., 2015).

Thus, we hypothesize that the effect of lysis on uninfected bacteria production and abundance, as well as on ecosystem functions, might depend on which nutrient (N or P) is limiting. In particular, we hypothesize that accounting for nutrient limitation furthers our understanding of the effect of phages on productivity and carbon sink, both of which vary with bacterial growth. Previous work has focused on the effect of marine phages on bacterial abundance and production, as well as on carbon and nutrient cycles, but it did not explicitly tackle the effect of nutrient limitation on the viral shunt. Here, we use a multitrophic model to compare the effect of lysis on a microbial system under N-limited and under P-limited conditions. Our model keeps track of the abundance of viruses, bacteria, zooplankton, and either N or P. With this model, we investigate the effect of lytic phages on (i) the abundance or concentration of organisms and nutrients in the system, with a focus on the bacterium-virus relationship, (ii) primary productivity, (iii) nutrient export to higher trophic levels, (iv) DOM release, and (v) carbon sink. Studying the carbon sink in this context allows us to explore the potential for using viral abundance as a predictor for the amount of carbon exported below the euphotic zone.

## Methods

**Figure 1:**
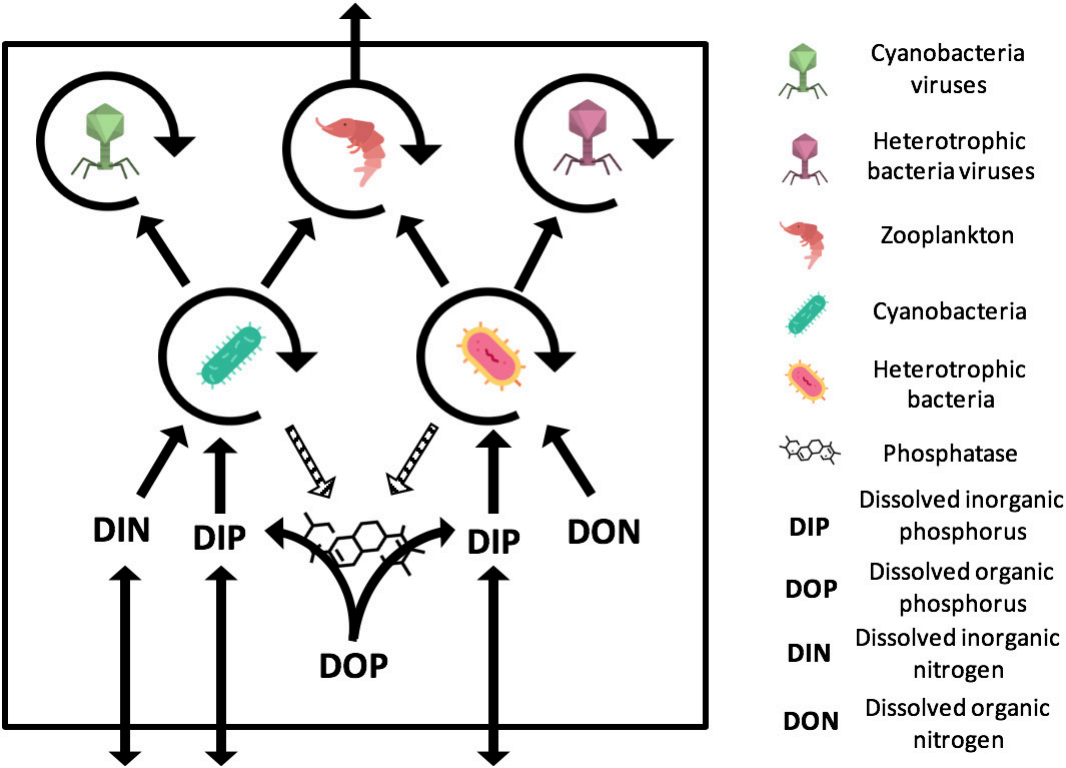
General representation of the N- and P-limited models with viruses. The virus-free models only differ from the absence of viruses. The N-limited model contains DIN and DON while the P-limited model contains DIP and DOP. Full arrows stand for gains or losses for a variable when pointed to or away from that variable. Arrow circles around a variable represent the multiplication of that variable. Dashed arrows are used for the secretion of phosphatase, which comes at no direct cost for bacteria. The release of inorganic and organic nutrients by bacteria, zooplankton, and viruses is not represented. Import or export out of the system are represented by arrows that intersect the square outline. Contributions to the carbon sink are not represented. Icons made by Freepik and Smashicons from www.flaticon.com.

We used a deterministic continuous-time (i.e., ordinary differential equation) modeling framework to explore the effect of phages on multitrophic systems in N- vs. P-limited conditions. The framework considers the interactions between bacteria, phages, zooplankton, and nutrients at the surface of the ocean, and includes exchanges with higher trophic levels and the subsurface (Figure 1). Bacteria, phages, and zooplankton have a fixed nutrient content, expressed in terms of the nutrient that is limiting. We parametrized the system using realistic ranges for each of the parameters, and constrained trait value combinations using well-known trade-offs found in the literature (see Parametrization). Each parameter set represented a realized microbial community, including its environment. The compilation of the outcome from several communities provided information to compare the emergent trends in N- vs. P-limited systems. We compared the outcome of the models both in the presence and in the absence of viruses.

### Model Description

Both our N-limited and our P-limited models include heterotrophic bacteria (H) and their viruses (V_H_), cyanobacteria (C) and their viruses (V_C_), zooplankton (Z), dissolved nutrients in inorganic form (N_in_ or P_in_), and dissolved nutrients in organic form (N_org_ or P_org_). This simplified representation of the microbial loop is similar to the one introduced by Weitz et al. (2015) to study the N-limited case only, except that we omit eukaryotic autotrophs, which is justified by the typical dominance of prokaryotic phytoplankton in oligotrophic environments (Follows et al., 2007). See Appendix A for model equations.

The P-limited version of our model differs structurally from the N-limited version only with regard to which form of the nutrient can cells take up. Here, we consider nutrient limitation in terms of the Liebig's law of the minimum (Baar, 1994), which is typically implemented by calculating the growth rates associated with each nutrient independently, and subsequently choosing the smallest to set the growth of the population. This differs from experimental approaches, which typically identify a nutrient as limiting when it falls below its detection limit (e.g., 0.05 μmol/L Graziano et al., 1996; Maat and Brussaard, 2016 for nitrate and 0.01 μmol/L for phosphate Maat and Brussaard, 2016). Both approaches are connected, as the expectation is that (i) a population will draw down the most limiting nutrient as much as it possibly can to utilize it for growth, and (ii) the nutrient that shows the lowest concentration will produce the lowest growth rate.

In both versions of our model, bacteria take up and assimilate nutrients following a Monod (i.e., saturating) function with parameters *r* (maximum growth rate), *K* (half-saturation constant) and ε (efficiency) (Monod, 1950). However, in the N-limited version, heterotrophic bacteria only use organic N (Equations A1 and A7), whereas cyanobacteria can only use inorganic N (Equations A2 and A6). In the P-limited system, both heterotrophic bacteria and cyanobacteria take up only inorganic P for growth and secrete phosphatase in the water to degrade organic phosphorus into its inorganic form (Equations A8, A9, and A14). In both versions, a fraction of the nutrient assimilated by bacteria is lost to respiration at a rate λ. An additional basal exudation rate (σ) represents the loss of organic material.

Zooplankton grow by consuming bacteria following a linear functional response with predation rate γ (Equations A3 and A10). Only a fraction of nutrients is used for growth, encoded with the efficiency parameter *p*_*g*_. A fraction *p*_*ex*_ is released as biomass and the rest is lost to the inorganic nutrient pool through respiration. In addition, zooplankton have a constant basal respiration rate, λ_*Z*_.

Viruses produce a fixed amount of progeny (burst size, β), virions that are released at a constant lytic rate, ϕ (Equations A4, A5, A11, and A12). The number of infections is proportional to the bacterial and viral concentrations. Note that, in this model, we do not consider lysogeny, as it does not contribute directly to the viral shunt. Because we assume that all infections result in direct lysis, infected bacteria are not included as an explicit class in this model. Upon lysis, the intracellular nutrients that were not used for virion production are released in organic form (Equations A7 and A14). Free viruses decay at a constant rate θ.

Nutrient import into the system occurs through an exchange of inorganic nutrients with the subsurface at a rate ω (Equations A6 and A13). Nutrients are exported from the system through the consumption of zooplankton by higher trophic levels (larger zooplankton and fish), encoded following a density-dependent predatory term (i.e., predation rate proportional to the squared density of zooplankton biomass). Part of this consumption is exported as biomass (at a rate *p*_*ex*_), and the rest is lost to the subsurface in the form of fecal pellets. In order to keep the model tractable, we assumed that all organic matter produced by the modeled zooplankton contribute to DOM (Equations A7 and A14) and can be ultimately reused by bacteria, whereas fecal pellets produced by zooplankton predators contribute to the carbon sink.

The variables and processes allowed us to define and monitor four fluxes, namely productivity, nutrient release, nutrient export to higher trophic levels, and carbon sink. We defined productivity as the total amount of biomass created by heterotrophic bacteria and cyanobacteria (Equation A15). Nutrient release represents the dissolved organic nutrients released by zooplankton and viruses through sloppy feeding and lysis, respectively (Equation A16). The predation term for zooplankton is used for nutrient export to higher trophic levels (Equation A17). Finally, carbon sink is the sum of the refractory carbon produced during lysis and of the sinking fraction of fecal pellets produced by predators of zooplankton (Equation A18). See Appendix A for their corresponding equations.

### Parametrization

Aiming to simulate the response of different microbial communities, we used a range of values to parametrize our equations. A literature search provided the minimum and maximum values possible to define a realistic range for each parameter (Appendix C, Table C-1). While the models presented here are general, parameters were selected when possible to represent the conditions in the North Atlantic Ocean—where both N and P limitation can be observed (Ammerman et al., 2003; Mather et al., 2008). Within these ranges, we used a Latin Hypercube Sampling (LHS) method on the log-transformed parameter ranges to generate random parameter sets (McKay et al., 1979). To constrain the trait combinations to realistic ones, we imposed well-known trade-offs for both bacterial and viral traits. For bacteria, we distinguished between fast-growing bacteria (i.e., high maximum growth rate, high half-saturation constant, and low phosphatase production) and slow-growing bacteria (low maximum growth rate, low half-saturation constant, and high phosphatase production) (Klausmeier et al., 2004; Litchman et al., 2007). Viruses were split between large viruses (high adsorption rate, low burst size, and low decay rate) and small viruses (low adsorption rate, high burst size, and high decay rate) (Bongiorni et al., 2005; Weitz, 2015). The range of each constrained parameter was approximately divided in half in log space, with the upper and lower half representing “high” and “low” values, respectively. For each parameter set, we randomly selected one type of bacteria (fast- or slow-growing) and viruses (small or large) and sampled from the corresponding restricted ranges. See Analysis for more details. We did not constrain the host-virus pairings (i.e., assumed no specificity) but, as with the rest of realized communities, we assumed instead as a realistic outcome any equilibrium for the system with coexistence among all organisms.

### Analysis

In order to characterize the realized behavior of each community, we solved analytically the system of equations composing the model to obtain the symbolic equilibrium expressions of each variable (Appendix B). In addition, and using the constraints explained in the previous section, we generated 1,000 random parameter sets (listed in Table C-1), each one representing a community. For each of these parameter sets, we then evaluated the equilibrium expressions, which we used to find the percentage of coexisting communities. In parallel, we also generated 500 random parameter sets to use as initial points for an optimization procedure: following Weitz et al. (2015), we applied a constrained optimization algorithm to find parameter values (within the imposed ranges) that minimized the difference between the models' outputs and values found in the field (Table 1). This optimization algorithm calculates the gradient of a specified function (in this case, a sum of the weighted differences between variable target values and values from the model) at a point in the parameter space, and aims to find the combinations of parameters that minimize this function. To this end, the algorithm takes steps in the direction of the largest negative gradient that are of magnitude proportional to this largest gradient, until a local minimum is reached. See the MATLAB documentation for the *fmincon* function for further details. We only used the resulting parameter sets if the algorithm found a minimum within the maximum number of steps that were allocated. The optimized parameter distributions across communities are presented in Figure C-1. We then evaluated our analytical expressions using these optimized parameter sets to find the associated equilibrium values for the different variables, and conserved only the parameter sets that resulted in coexistence with and without viruses. Note that this optimization algorithm is computationally expensive, reason why we reduced the number of replicates with respect to the ones generated to study the percentage of coexistence. These two approaches to generate realistic communities allowed us to study the role of viruses for both the N- and P-limited versions of the model. Because the P-limited system without viruses did not allow for coexistence between heterotrophic bacteria and cyanobacteria (see below), we limited the P-limited virus-free version of the model to heterotrophic bacteria only.

**Table 1:** Variable target values for optimization.

**Variable (Model abbreviation)**	**Target value**	**Units**	**References**
Heterotrophic bacteria (H)	6 × 10^8^	Particles/L	Li, 1998
Cyanobacteria (C)	1 × 10^8^	Particles/L	Johnson et al., 2006
Zooplankton (Z)	4 × 10^4^	Particles/L	Schartau et al., 2010
Heterotrophic bacteria viruses (V_H_)	9 × 10^9^	Particles/L	Suttle, 2007
Cyanobacteria viruses (V_C_)	1.5 × 10^9^	Particles/L	Suttle, 2007
Dissolved inorganic nitrogen (N_in_)	0.1	μM	Shelford et al., 2012
Dissolved organic nitrogen (N_org_)	5	μM	Letscher et al., 2013
Dissolved inorganic phosphorus (P_in_)	7 × 10^−3^	μM	Mather et al., 2008
Dissolved organic phosphorus (P_org_)	0.1	μM	Mather et al., 2008

In addition to each dynamic variable (bacterial, viral, zooplankton, and organic and inorganic nutrient densities), we also evaluated fluxes of interest: productivity, organic nutrient release, export to higher trophic level, and carbon sink. For each equilibrium with coexistence, we assessed stability by using MATLAB to find numerically the eigenvalues of the Jacobian matrix evaluated at that equilibrium. This exercise allowed us to distinguish between stable nodes (real negative eigenvalues), unstable nodes (at least one real positive eigenvalue), stable spirals (complex eigenvalues with negative real part) and unstable spirals (complex eigenvalues, at least one of which shows a positive real part), the last two being characterized by oscillations around the equilibrium value.

Finally, we used non-optimized equilibrium values to study the relationship between viruses and bacteria, and between viruses and carbon sink. Including non-optimized equilibria allowed for a large diversity of equilibria. Because the expectation for the relationship between bacteria and viruses is a power law i.e., *V* = α*B*^γ^ (Wigington et al., 2016), we calculated the coefficient of the power law that best described this relationship in our simulations. The power law coefficient corresponds to the slope γ of the regression between bacteria and viruses in log space:

(1) $$\begin{array}{r} logV=log\alpha +\gamma logB \end{array}$$

In order to obtain an ensemble of regression coefficients, we repeated this process a certain number of replicates. More specifically, we performed 50 linear regressions between bacteria and viruses that used the coexisting communities/equilibria resulting from 200 random parameter sets each (~70 coexisting communities/equilibria per replicate/linear regression). This protocol allowed us to get an average value for the slope and coefficient to ensure that the power law coefficients were representative.

We used a similar procedure to describe the relationship between viruses and carbon sink.

## Results and Discussion

### Coexistence and Stability

**Figure 2:**
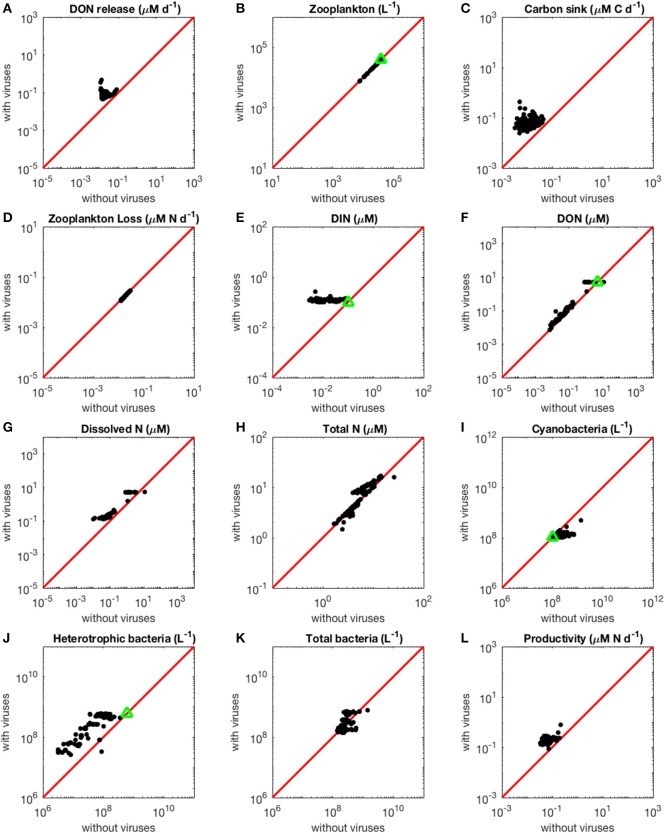
Effect of viruses on steady-state concentrations and fluxes for the nitrogen-limited system. The red line denotes the 1:1 line and the green triangles show target densities used in the optimization procedure. Each point stands for the steady concentration for one optimized parameter set. Points above and below the red line represent steady-state values that increased and decreased with viruses, respectively. **(A)** Release of dissolved organic nitrogen by zooplankton and viruses. **(B)** Zooplankton abundance. **(C)** Carbon sink. **(D)** Export of nitrogen to higher trophic levels through zooplankton predation. **(E)** Dissolved inorganic nitrogen. **(F)** Dissolved organic nitrogen. **(G)** Total dissolved nitrogen (DIN and DON). **(H)** Total nitrogen (dissolved and organismal). **(I)** Cyanobacteria abundance. **(J)** Heterotrophic bacteria abundance. **(K)** Total bacterial abundance (heterotrophic bacteria and cyanobacteria). **(L)** Biomass production by heterotrophic bacteria and cyanobacteria.

With the N-limited version of the model, coexistence of all populations occurred in 87 out of 1,000 communities (8.7%) for random parameter sets. This number only refers to parameter sets that led to coexistence when used both with and without viruses. As a comparison, coexistence occurred in only 0.45% of randomly generated communities in a similar model comprising an additional class of eukaryotic phytoplankton (Weitz et al., 2015). The omission of autotrophic eukaryotes in our model thus facilitated coexistence, as expected given the reduction in the competition experienced by cyanobacteria. When we evaluated the steady-state concentrations obtained for parameters resulting from the optimization algorithm, coexistence emerged in 131 out of 500 communities (26.2%; see resulting stationary values in Figure 2). Out of these 131 coexisting communities, 70 (53.4%) contained fast-growing heterotrophic bacteria, while the remaining 61 communities were composed of slow-growing heterotrophic bacteria. Approximately 45% of the 131 communities harbored fast-growing cyanobacteria. In addition, 58% of the 131 communities were composed of large viruses that infect heterotrophic bacteria, while 55% of them were composed of large cyanobacteria-infecting viruses. Viruses infecting heterotrophic bacteria were the only group to show a significantly different distribution after optimization and selection for coexistence, compared to the original random growth rate distribution (Chi-squared test, *p* = 0.0338). There were no significant correlations between the growth rates of heterotrophic bacteria and cyanobacteria (Pearson's correlation test, *p* = 0.0522), or between the sizes of the two types of viruses (Pearson's correlation test, *p* = 0.786). Similarly, there was no significant association between bacterial growth rate and the size of their infecting viruses (Pearson's correlation test, H: *p* = 0.400, C: *p* = 0.841).

**Figure 3:**
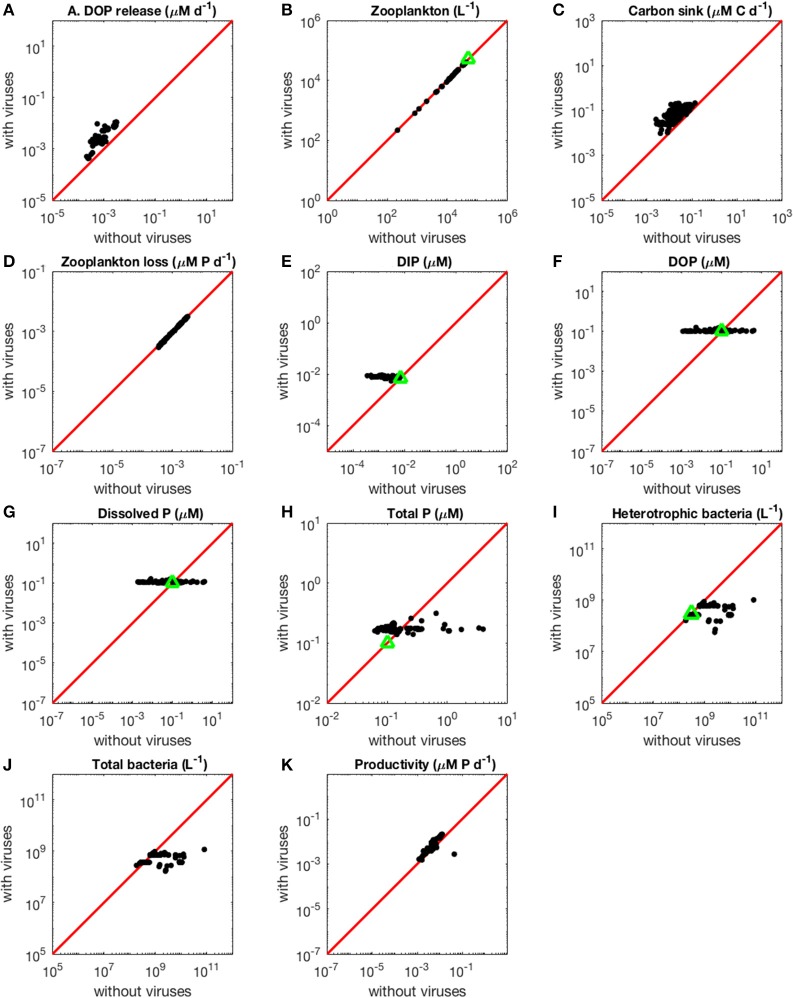
Effect of viruses on steady-state concentrations and fluxes for the phosphorus-limited system. Cyanobacteria are not represented because they are not present in the virus-free system. The red line denotes the 1:1 line and the green triangles show target densities used in the optimization procedure. Each point stands for the steady concentration for one optimized parameter set. Points above and below the red line represent steady-state values that increased and decreased after introducing viruses, respectively. **(A)** Release of dissolved organic phosphorus by zooplankton and viruses. **(B)** Zooplankton abundance. **(C)** Carbon sink. **(D)** Export of phosphorus to higher trophic levels through zooplankton predation. **(E)** Dissolved inorganic phosphorus. **(F)** Dissolved organic phosphorus. **(G)** Total dissolved phosphorus (DIP and DOP). **(H)** Total phosphorus (dissolved and organismal). **(I)** Heterotrophic bacteria abundance. **(J)** Total bacterial abundance (heterotrophic bacteria and cyanobacteria). **(K)** Biomass production by heterotrophic bacteria and cyanobacteria.

**Figure 4:**
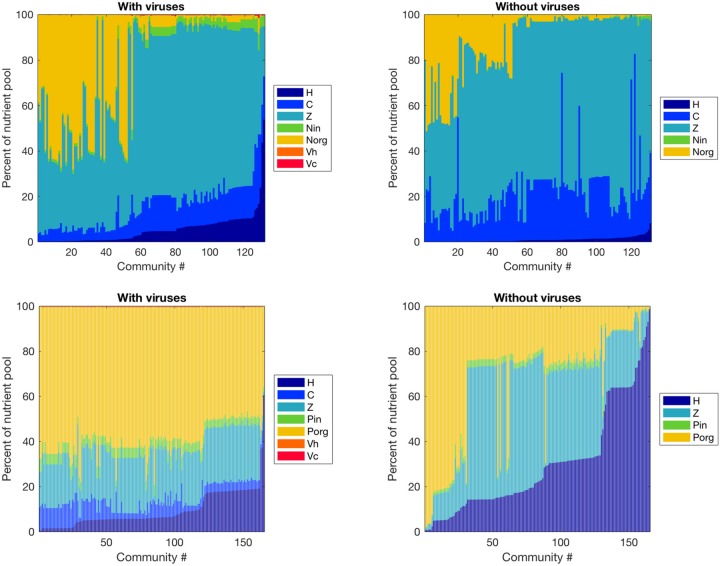
Effect of viruses on the partitioning of the nutrient pool between all variables. The nutrients stored in each variable were calculated with equilibrium values and parameters, and we obtained percentages by dividing by the total amount of nutrient in the system. **Top**: Nitrogen-limited model. **Bottom**: Phosphorus-limited model. Communities are ordered by ascending order of the percentage of the nutrient pool stored in heterotrophic bacteria.

In the virus-free P-limited system, coexistence between heterotrophic bacteria and cyanobacteria was not possible. Without viruses, both types of bacteria rely on the same resource and are preyed upon by the same class of zooplankton. As a consequence, and in agreement with classic resource competition theory, one bacterial class always outcompetes the other. The presence of host-specific viruses prevented competitive exclusion from happening, consistent with the hypothesis that viruses increase bacterial diversity (Thingstad, 2000). In order to make the model with and without viruses as comparable as possible, and noticing that when viruses are present heterotrophic bacteria dominate over cyanobacteria (i.e., have a higher median for abundance, see Table 2, and biomass, see Figure 4), we focused the P-limited case on heterotrophic bacteria only (Figure 3). Importantly, all results were qualitatively identical when using instead a cyanobacteria-only version of the virus-free model (Figure D-1). Out of 1,000 random parameter sets used for the P-limited case, 358 (35.8%) allowed for coexistence of all the populations originally present, both with and without viruses. Reducing the virus-free case to one bacterial class in the P-limited model removed the pressure of bacterial interspecific competition, and explained partially the high rate of coexistence in the P-limited case compared to the N-limited one (100 and 92.8%, respectively). However, the percentage of coexistence was also different between the N- and P-limited versions with viruses (8.7 and 35.8%, respectively), suggesting that intrinsic differences like the possibility to take up also the organic form of the nutrient play a role. Following optimization, we obtained coexistence in 165 out of 500 runs (33%; see resulting stationary values in Figure 3), a percentage that is not significantly different from that of the random sampling (Chi-square test, *p* = 0.283). This is in contrast with the ~3-fold boost obtained in the N-limited case when using the optimization algorithm, and suggests that the optimization procedure was not significantly more successful than the random sampling in finding feasible equilibria for the P-limited case. Moreover, for some of the 500 optimized parametrizations the optimization function failed to find an optimum in the allocated number of steps, which suggests the existence of regions with low gradient in the parameter space. Out of the 165 coexisting communities, 59 (35.8%) and 63 (38.2%) contained fast growing heterotrophic bacteria and cyanobacteria, respectively. Both of these proportions were significantly different from the random distributions (50.6 and 53.2%) prior to optimization (Chi-squared test, *p* < 0.001 for both). Coexistence thus occurred more frequently when bacteria grew slowly. On the other hand, the sizes of heterotrophic-bacteria-infecting viruses and cyanobacteria viruses in coexisting communities after optimization did not differ significantly from random distributions (Chi-squared test, *p* = 0.360 and *p* = 0.146, respectively). There were significant correlations between the sizes of viruses (Pearson's correlation test, *p* = 0.392) and between bacterial growth rate and the size of their infecting viruses (Pearson's correlation test, H: *p* = 0.453, C: *p* = 0.126). The growth rates of heterotrophic bacteria showed a significant negative correlation with the growth rates of cyanobacteria in coexisting communities (Pearson's correlation test, ρ = −0.456, *p* = 7.37e−10). In other words, coexistence occurred more frequently when one type of bacteria grew slowly and was efficient at harvesting nutrients, while the other grew faster and was less efficient at getting nutrients.

**Table 2:** Median for each variable and flux in N- and P-limited systems with and without viruses, and for the effect of viruses, corresponding to the ratio of the first over the other, respectively.

**Variables**		***N*-limited**			***P*-limited**	
	**With V**	**Without V**	**Effect of V**	**With V**	**Without V**	**Effect of V**
H (indiv. L^−1^)	2.67E+08	4.62E+07	6.40E+00	4.24E+08	6.42E+08	8.85E−01
C (indiv. L^−1^)	1.14E+08	2.02E+08	5.68E−01	1.00E+08	NA	NA
Z (indiv. L^−1^)	3.83E+04	3.90E+04	9.81E−01	3.91E+04	3.97E+04	9.81E−01
N_in_ or P_in_ (μM)	1.13E−01	7.60E−03	1.44E+01	7.10E−03	2.20E−03	3.42E+00
N_org_ or P_org_ (μM)	1.47E−01	8.73E−02	1.66E+00	1.00E−01	1.99E−02	5.04E+00
V_H_ (indiv. L^−1^)	1.50E+09	NA	NA	8.97E+09	NA	NA
V_C_ (indiv. L^−1^)	8.15E+09	NA	NA	1.49E+09	NA	NA
NR (μM day^−1^)	7.56E−02	1.65E−02	4.05E+00	5.10E−03	1.00E−03	3.56E+00
NE (μM day^−1^)	1.39E−02	1.46E−02	9.61E−01	1.70E−03	1.70E−03	9.62E−01
CS (μM day^−1^)	6.42E−02	1.25E−02	5.17E+00	8.06E−02	2.03E−02	3.00E+00
P (μM day^−1^)	1.84E−01	5.27E−02	3.83E+00	8.60E−03	4.90E−03	1.52E+00

*NR, NE, CS, and P correspond to nutrient release, nutrient export to higher trophic levels, carbon sink and productivity, respectively*.

A majority of coexisting equilibria were also stable. A community that reaches a stable equilibrium will be able to recover from small perturbations (mathematically, all the variables representing the system go back to their equilibrium value following small perturbations). In contrast, if the system is at an unstable equilibrium, it will continue to move away from it when perturbed. Thus, the (ins)stability of an equilibrium is directly related to the resistance and resilience of the community against external perturbations. Regardless of whether the system moves toward or away from equilibrium after a perturbation, this movement can either follow a monotonic path or an oscillatory one, in which case the equilibrium is called a node or a spiral, respectively. Thus, the system can show different degrees of temporal variability when responding to the perturbation. With viruses, we found 100 and 85.5% of stable spirals in the N- and P- limited systems, respectively. In the P-limited system, the remaining equilibria behaved as unstable spirals. Without viruses, stable spirals still dominated (N-limited: 77.9%, P-limited: 95.8%), but stable nodes were also present (N-limited: 7.6%, P-limited: 4.2%). The remaining equilibria in the N-limited system were unstable spirals. There were no unstable equilibria in the P-limited system.

### Effect of Viruses on the Ecosystem

#### The Increase in Nutrient Release With Viruses Is Larger in the N- Than in the P-Limited System

As expected from the description of the viral shunt, the release of organic nutrients in both N-limited and P-limited systems increased in the presence of viruses (Figures 2A, 3A). The presence of viruses led to a very small but consistent decrease in zooplankton abundance and a corresponding decrease in nutrient release by zooplankton in both the N- and P-limited systems (Wilcoxon signed rank test, *p* < 0.001 for both, Table 2). The decrease in zooplankton abundance was similar in the N-limited and the P-limited systems (Wilcoxon rank sum test, *p* = 0.173). However, this decrease of zooplankton contribution to nutrient release was small enough to be compensated for by the inclusion of viruses, leading to an overall increase in nutrient release that was larger in the N-limited system than in the P-limited system (Wilcoxon rank sum test, *p* = 0.0168, Figures 2A, 3A). Nutrient release from lysis was affected both by total bacterial abundance and by the amount of nutrients released per lysed bacterium. Total bacterial abundance increased in the presence of viruses in half of the N-limited communities but consistently decreased in the P-limited system, explaining in part the larger increase in nutrient release observed in the N-limited system. In order to control for bacterial abundance and isolate the effect of the stoichiometric mismatch between bacteria and viruses, we explicitly evaluated the amount of nutrient released per bacterium during lysis at optimized parameter sets and variable values. On average, each lysed heterotrophic bacterium released 2.04 × 10^−10^ μM out of the 5.98 × 10^−10^ μM of N it initially contained in the N-limited model, equivalent to a release of 34.2% of the bacterial N content. If that total cell content were used for viral production, it would produce 487 virions on average. In contrast, 8.14 × 10^−12^ μM (21.2%) out of 3.84 × 10^−11^ μM of P was released in the environment in the P-limited model, and a heterotrophic bacterium contained on average enough P to make 383 virions. Thus, each progeny virus will take up more of the limiting nutrient in the P- than in the N-limited system. The medians were lower in both cases (426 and 347, respectively), showing a skew to the left of these bacteria-to-virus nutrient ratios. The differential release of N and P between the two systems is thus explained in part by the nutrient requirement of the virus: for a fixed number of viral progeny, proportionally more N than P is unused by the viruses and therefore released at lysis. A lower proportion of nutrients was released from cyanobacteria (N: 2.19 × 10^−10^ out of 3.88 × 10^−9^ μM or 5,7%; P: 4.57 × 10^−12^ out of 9.93 × 10^−11^ or 4,6%), which can be explained by the higher burst size of cyanobacteria-infecting phages (Table C-1). Overall, these observations confirm that the difference in the magnitude of nutrient release between the N- and P-limited systems at the community level can be traced to differences in the nutrient content of bacteria and viruses.

The high N:P ratio of the lysate we observed across the two versions of our model is consistent with the predictions made by Jover et al. (2014) using C:N:P ratios for three different cyanobacteria, while the exact proportions of both N and P were higher in our model than in their predictions. The latter were made using a burst size of 40, which is at the lower end of the range used in this model (Table C-1), and explains this difference. In nature, burst size would be limited by the size, physiology, and nutrient content of the bacterium (Parada et al., 2006; Choua and Bonachela, 2019), which cannot be implemented in our model due to the fixed nutrient content of bacteria. Including this effect would likely constrain the proportion of nutrients released between the estimate from Jover et al. (2014) and ours.

#### The Contribution to the Carbon Sink Increases More in the N-Limited than in the P-Limited System in the Presence of Viruses

Total carbon exported due to sinking debris increased in the presence of viruses (Figures 2C, 3C), which could be explained following a similar reasoning as for nutrient release. The contribution from zooplankton consumption to the carbon sink only decreased slightly, whereas lysis generated a large amount of carbon. We found carbon sink values between 10^−2^ and 5 × 10^−1^ μM carbon per day in both N- and P-limited systems. This is equivalent to a range of 2.2–109.5 g of carbon per m^2^ per year. For the conversion, we assumed a euphotic zone depth of 50 meters, corresponding to the depth at which nutrient concentration starts to increase in the Atlantic (Ammerman et al., 2003). By comparison, local sediment traps at different locations in the Atlantic Ocean recorded carbon sink rates of 0.4 to 4 g carbon per m^2^ per year (Antia et al., 2001). Using a similar method, Marsay et al. (2015) found sinking rates between 15 and 30 g POC per m^2^ per year at a depth of 100 m, which decreased due to remineralization to rates below 3 g POC per m^2^ per year at a depth of 1,000 m. MEDUSA (Yool et al., 2013), a biogeochemical model of the marine carbon cycle, provides similarly low estimates of carbon sink rates, below 7.5 g carbon per m^2^ per year in most pelagic regions of the North Atlantic (Barange et al., 2017). The range from our model overlaps with the empirical one, showing that at least some of the steady-state concentrations we obtained are consistent with realistic carbon sink values. The higher values obtained from our model could be explained by variation in the effective euphotic zone depth in the field, and/or by the fact that our carbon sink rates were calculated at the bottom of the euphotic zone while traps are placed hundreds or thousands of meters deep. Deeper traps tend to collect less particulate organic products than shallower ones, supporting the latter hypothesis (Antia et al., 2001). This difference can be attributed to remineralization during transport to the bottom of the ocean, for which we did not account. The increase in carbon sink was larger in the N-limited than in the P-limited system (Wilcoxon rank sum test, *p* < 0.001). In our model, carbon sink is determined by zooplankton fecal pellets and debris from lysis, the latter being in turn proportional to bacterial and viral abundances. We did not find a difference in the magnitude of the decrease of zooplankton concentration in the presence of viruses, and the total abundance of viruses was larger in the P-limited system (Wilcoxon rank sum test, *p* < 0.001). The larger increase in carbon sink in the N-limited system must then result from a larger positive change in bacterial abundance in the N-limited than in the P-limited system.

#### Loss of Zooplankton Results in an Increase in Inorganic Nutrients With Viruses

The small decrease in zooplankton concentration with viruses also resulted in a similar decrease in zooplankton predation and nutrient export to higher trophic levels (Table 2). The decreased export affected indirectly the dissolved inorganic form of nutrients (Figures 2E, 3E). Inorganic nutrient concentrations increased in both systems. The magnitude of this increase was larger in the N-limited system (Wilcoxon rank sum test, *p* < 0.001). In our model, exchange with the subsurface is determined by the difference between inorganic nutrient concentration at the surface and that at the subsurface. If inorganic nutrient concentration is higher at the surface, then a net export of nutrients from the surface to the subsurface occurs. Conversely, a higher nutrient concentration in the subsurface results in a net import into the system. Loss of zooplankton and exchange with the subsurface are the only sources of N and P exchange with the outside of the system in our model (Figure 1). Because the total nutrient pool (i.e., total organic and inorganic nutrient, either N or P, within the system) is constant at equilibrium, the net export represented by zooplankton removal must be balanced by a net import from the subsurface. At equilibrium, the nutrient export decrease associated with viruses is balanced by a matching nutrient import decrease. This means that, in order to observe a low nutrient import, the difference between the surface and the subsurface must be smaller, i.e., the inorganic nutrient concentration at the surface has to be large to match the higher concentration in the sub-surface (Equations B4, B14, B21, and B32). Note that dissolved organic nutrients are not affected in this way because our model omits the exchange of organic nutrients with the sub-surface. If the latter were present, we would observe a similar phenomenon but with respect to the sum of inorganic and organic nutrients. Overall, including exchanges with the sub-surface for all other variables would provide an additional import of nutrients and an export of bacteria, zooplankton and viruses.

The changes in organic nutrients resulting from the presence of viruses differ for N- and P-limited systems (Figures 2F, 3F). In the P-limited system, DOP increased in 81% of communities, with the rest showing a decrease. Because DOP concentrations were at least an order of magnitude higher than DIP concentrations, the total dissolved phosphorus (DIP + DOP) showed a similar behavior (Figure 3G), as well as the total phosphorus pool, which includes DIP, DOP, and organismal P (Figure 3H). In N-limited systems, on the other hand, DON always increased when viruses were included (Figure 2F), leading to a similar trend for total dissolved nitrogen (Figure 2G) and the total nitrogen pool (Figure 2H).

#### Bacterial Abundance at Equilibrium is Top-Down Regulated by Viruses, Leading to an Increase and Decrease in the N- and P-Limited Systems Respectively

Although it is well-known that nutrient concentration affects the bacterial nutrient uptake rate (implemented in our model through Equations A1, A2, A8, and A9), here we did not observe any effect on the abundance of bacteria at steady-state (Figures 2I–K, 3I,J). Without viruses, the bacterial abundance is a function of zooplankton abundance, in turn determined by a range of parameters relating to bacteria metabolism and nutrient exchange with the subsurface (Equations B16, B17, and B31). In contrast, when viruses are present, bacterial abundances are completely set by the three parameters describing viral dynamics—burst size, adsorption rate and decay rate, i.e., bacteria are purely top-down controlled by their corresponding viral population (Equations B1, B2, B18, and B19). In other words, the indirect bottom-up control of uninfected bacteria that viruses exert through nutrient release during lysis can impact transient dynamics but does not determine equilibrium values.

In the N-limited system, and in agreement with (Weitz et al., 2015), these dependencies resulted in a dichotomy between cyanobacteria, whose abundance consistently decreased when viruses were present (Figure 2I), and heterotrophic bacteria, whose abundance always increased (Figure 2J). This difference cannot be attributed to a single cause: heterotrophic bacteria and cyanobacteria differ in terms of their viral-dynamics parameters (affecting their abundance with viruses) and of their uptake dynamics (affecting their abundance without viruses). In contrast, in the P-limited system the abundance of heterotrophic bacteria decreased when viruses were introduced (Figure 3I). This is expected, as the virus-free version lacks both mortality due to viruses and competition with cyanobacteria. We cannot compare the abundance of cyanobacteria with and without viruses because cyanobacteria were not present in our P-limited virus-free model. The total abundance of bacteria (H without viruses, H plus C with viruses) decreased when viruses were introduced despite the inclusion of cyanobacteria in the system with viruses (Figure 3J). This discrepancy between the N- and P-limited systems cannot be explained by the bacterial concentrations in the presence of viruses, because these are entirely determined by the virus-related parameters, which were similar between the N- and P-limited systems. Therefore, the difference between the N- and P-limited systems must originate from the virus-free dynamics. This hypothesis is confirmed by a comparison of bacterial abundances in the virus-free systems: total bacterial abundances in the virus-free P-limited system are an order of magnitude higher than the total bacterial abundances in the virus-free N-limited system (Figures 2K, 3J). In summary, total bacterial abundance is comparable between the N- and P-limited systems when top-down controlled by viruses, but total bacteria abundance is sustained at a higher level in the P-limited system than in the N-limited system when zooplankton is the source of top-down regulation.

Even though total bacterial abundance decreased in most communities in the P-limited system, productivity still increased in the majority of them (Figures 2L, 3K). This increase results from the increase in nutrient concentration in the presence of viruses. In other words, the decrease in bacterial abundance was compensated by the increase in nutrients, leading to faster growth. The increase in productivity was larger in the N-limited than in the P-limited system (Wilcoxon rank sum test, *p* < 0.001), following the larger increase in nutrient release.

#### Nutrient Pool Partitioning is More Affected by Viruses in the P- than in the N-Limited System

Viruses affected the proportion of nutrients stored as bacteria, zooplankton, and dissolved nutrients differently in the N- and P-limited systems (Figure 4). In the N-limited system, the bacterial nutrient pool—the total amount of nutrients stored in all bacteria—was dominated by cyanobacteria in the absence of viruses, and was split equally between heterotrophic bacteria and cyanobacteria when viruses were present. This resulted from the decrease in cyanobacteria abundance and increase in heterotrophic bacteria abundance observed with viruses. In total, bacteria represented a minimum of 5% and a maximum of 30% of the total nutrient pool (including dissolved and intracellular nutrients) both with and without viruses, in agreement with the absence of a clear effect of viruses on total bacteria abundance. For 50% of communities, this increase in nutrients stored in heterotrophic bacteria was the only noticeable change, and the nutrient partitioning between bacteria, zooplankton, and dissolved nutrients was conserved. For the other 50%, the zooplankton share of the total nutrient pool decreased in favor of dissolved organic nitrogen when viruses were included. In contrast, the P-limited system was characterized by a consistent decrease in the proportion of nutrients stored in bacteria and zooplankton in the presence of viruses, with a corresponding increase in DOP. In addition, cyanobacteria represented a smaller share of the total nutrient pool in the P-limited than in the N-limited system. The large share of the nutrient pool taken up by heterotrophic bacteria in the virus-free P-limited system is consistent with our previous observation on bacterial abundance: heterotrophic bacteria tend to fare better in a P-limited than in a N-limited system when viruses are absent. In both systems, the zooplankton share generally decreased, matching the small but significant decrease in abundance described above. It is interesting to note that viruses themselves represented a negligible proportion of the total nutrient pool. This suggests that their large influence on nutrient cycles depends on a high number of lysed bacteria rather than a high nutrient content.

### Virus-to-Prokaryote Ratio

**Figure 5:**
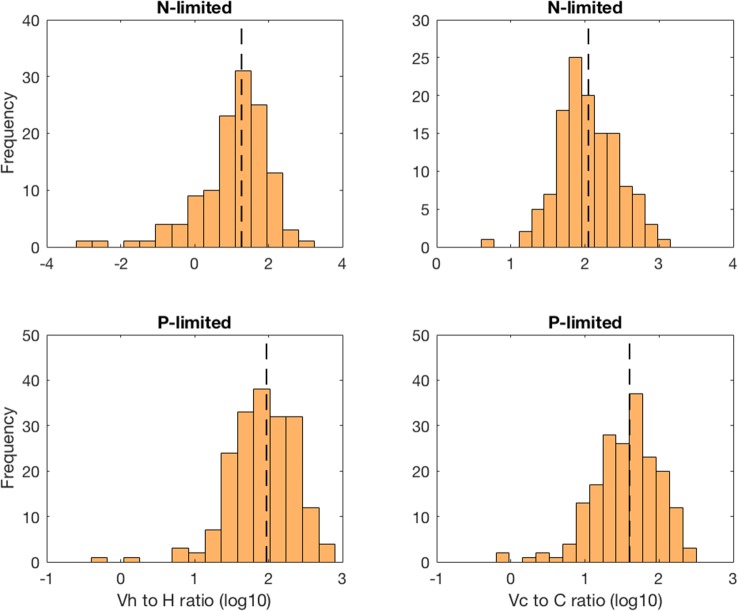
Distribution of the virus-to-prokaryote ratio in log space for heterotrophic bacteria (left column) and cyanobacteria (right column) in the N- and P-limited models. The dashed lines represent the median for each distribution.

The virus-to-prokaryote ratio (VPR) spanned multiple orders of magnitude in both N- and P-limited versions of the model, and for both heterotrophic bacteria and cyanobacteria (Figure 5). We observed the most diversity in VPR across communities for heterotrophic bacteria in the N-limited system, where the ratio ranged from 0.001 to 1000 with a median close to 10. We applied a log transformation to VPRs to reduce the skew of the data. The standard deviation was 0.98 in log space, corresponding to nearly an order of magnitude. In contrast, the VPRs for cyanobacteria were confined between 10 and 1000 with a median just above 100 and a standard deviation of 0.41 in log space (equivalent to a factor of 2.6). In the P-limited system, the VPR distributions for heterotrophic and cyanobacteria were similar to each other, with most of the communities having a VPR between 1 and 300, a median close to 30 and 100, respectively, and a standard deviation of 0.44 for both (equivalent to a factor 2.8). Although the consensus until recently was that viruses outnumbered bacteria 10 to 1 (i.e., constant VPR = 10), with typical respective abundances of 10^9^ and 10^8^ particles per liter (Bergh et al., 1989; Chibani-chennoufi et al., 2004), recent VPRs gathered from hundreds of studies of marine pelagic environments ranged from 0.0075 to 2150, with a mean of 26.5 (Parikka et al., 2017). Thus, variation of parameters within realistic ranges—representing a diversity of microbial communities and environmental conditions—allowed for wide ranges of VPRs that are consistent with the most current field observations.

**Figure 6:**
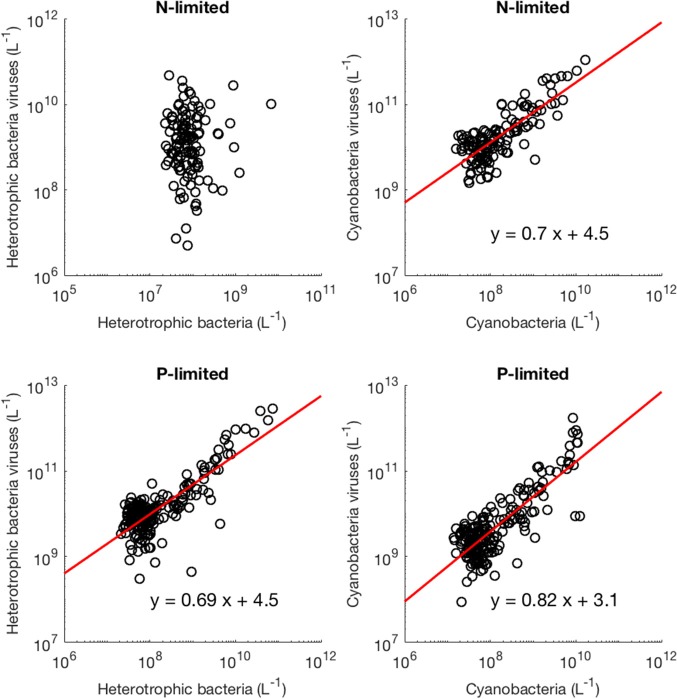
Relationship between viruses and bacteria. The red line represents the best-fit line obtained through a linear regression in log-space. The best-fit line was not included if bacterial abundance was not a better predictor of viral abundance than the mean of viral abundance (intercept-only model).

**Figure 7:**
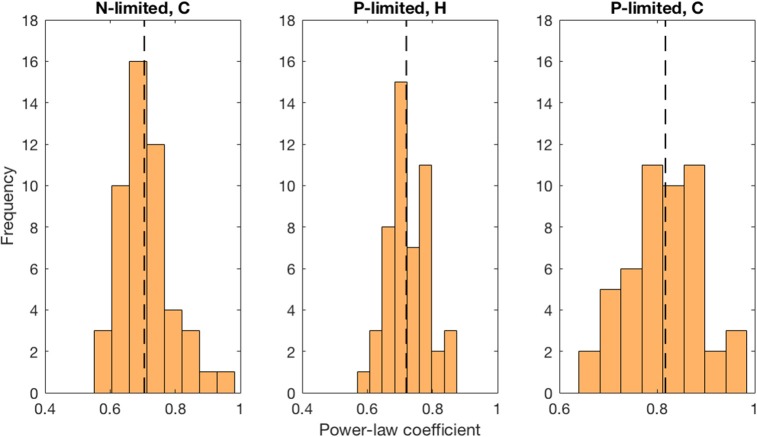
Distribution of best-fit power-law coefficients. We used more than 50 communities for each linear regression and repeated 50 times. Each power-law coefficient corresponds to the slope of a linear regression in log-space. The dashed line represents the median for each distribution.

Moreover, we found that viral abundance followed a power law as a function of bacterial abundance with an exponent below 1 for both types of bacteria in the P-limited system (H: slope = 0.691, *p* < 0.001, *R*^2^ = 0.642; C: slope = 0.817, *p* < 0.001, *R*^2^ = 0.68, Figure 6), and for cyanobacteria in the N-limited model (slope = 0.702, *p* < 0.001, *R*^2^ = 0.635). In other words, an increase in bacteria was associated with a less-than-proportional increase in viruses. A power law was not a meaningful representation of the relationship between the concentration of heterotrophic bacteria and their viruses in the N-limited case (F-statistic = 0.0246, *p* = 0.876, *R*^2^ = 1.96 × 10^−4^). Interestingly, heterotrophic bacteria in the N-limited system are the only ones to be limited by an organic nutrient in our model. Both types of bacteria only take up inorganic P, but cyanobacteria only use inorganic N. It is unclear how reliance on inorganic nutrients would result in a significant power-law relationship between bacterial and viral abundance. After generating an additional 50 linear regressions with at least 50 different surviving communities each, the majority of the slopes were between 0.6 and 0.9 (Figure 7), which corroborated the results discussed above. These results are consistent with Wigington et al. (2016), who analyzed data from surveys around the world and found that the relationship between viruses and bacteria is better described by a sublinear power-law than by a linear model.

### Relationship Between Viruses and Carbon Sink

**Figure 8:**
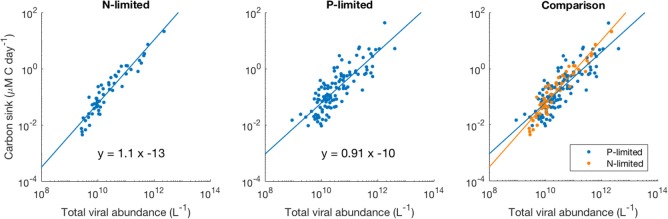
Relationship between viral abundance and carbon sink in the N- and P-limited models. The lines represent the best-fit line obtained through a linear regression in log-space. R^2^ were 0.89 and 0.68 for the N- and P-limited system, respectively.

We observed a four-order-of-magnitude variation across communities in the values of carbon sink that emerged from the model. Variation over multiple orders of magnitude of carbon flux across the world has also been observed empirically, especially close to the surface (Mouw et al., 2016). Importantly, in our N-limited and P-limited systems total viral abundance alone explained 89 and 68% of the carbon sink variation observed with our model, respectively (Figure 8). The linear regressions for both systems were very similar, which suggests that local nutrient limitation does not play a relevant role in this relationship. The associated error differed between the N- and P-limited cases but stayed relatively small for both systems, compared to the variation of the carbon sink values. In the N-limited system, the root mean square error (RMSE) was equal to 0.283 in log-space. This number, which cannot be converted to a single number out of log space (the error is exponential and depends on the predicted value), corresponds to a mean error of 90% above the predicted value and 48% below the predicted value. For a carbon sink of 0.1 μM of carbon per day, this corresponds to a mean error of 0.09 above and 0.048 below 0.1 μM of carbon per day. In the P-limited system, the RMSE was equal to 0.429, corresponding to an error of 168% above and 62% below the predicted value.

### Framework Limitations and Simplifications

For this work, we decided to focus on N and P limitation based on the stoichiometric mismatch between bacteria and viruses, which could affect the proportion of nutrients released during lysis (Jover et al., 2014). However, P limitation and N limitation are only common in subtropical regions. At higher latitudes, iron limitation becomes widespread (Moore et al., 2002; Mather et al., 2008). In addition, certain organisms have specific nutrient requirements, such as diatoms that can be limited by silica (Leynaert et al., 2001; Brzezinski et al., 2003). To understand the effect of viruses globally, we would then need to include these other nutrients in our model. In this context, a more realistic understanding of the system would require including both nutrients simultaneously, potential interactions between them, and the possibility for co-limitation (Bonachela et al., 2015). How to best model both nutrients simultaneously is still an open problem, and beyond the scope of this work. In addition to considering that a single nutrient is limiting at any given time, we made two important simplifications in order to reach analytical expressions for the steady state. First, we did not include a class of infected cells. Depending on the phage and its host, the period of infection can vary from minutes to days (Weitz, 2015), time during which both infected host and virus show a behavior that differs from their free counterparts. This is important because viral genes manipulate the metabolism and nutrient uptake of infected bacteria to facilitate the production of progeny viruses (Zeng and Chisholm, 2012; Puxty et al., 2016), and progeny synthesis in turn depends on the physiological state of the infected host (Hadas et al., 1997; You et al., 2002; Choua and Bonachela, 2019). Furthermore, we determined a fixed nutrient content for each organism, which does not represent accurately the wide variation of cellular elemental composition observed depending on nutrient availability and other environmental conditions (Bonachela et al., 2015). This would, of course, affect the amount of nutrient released during lysis but also, e.g., nutrient uptake dynamics.

Despite these limitations, our model yields variable values consistent with those observed in the field (Table 1) when realistic parameter values are used. In addition, we showed that at least two of its emergent properties (carbon sink and VPR) are within the range observed in empirical studies.

## Conclusion

Marine phages play a unique role in microbial communities through the combined effect of the release of nutrients left over from virion production, and bacterial mortality (Suttle, 2007). Because viral lysis releases nutrients with a higher N:P ratio than that of bacteria (Jover et al., 2014), we hypothesized that the effect of phages on microbial communities would depend on nutrient limitation. Using N- and P-limited multitrophic models, we found a clear influence of nutrient limitation on the magnitude of nutrient release, which translated into a difference in the effect of viruses on productivity between the two versions of the model. In contrast, the steady-state values of some variables—zooplankton, export to higher trophic levels and inorganic nutrients—were not affected by this nutrient release but rather by the additional mortality in the model with viruses. Unlike the effect of nutrient release, the impact of this mortality is independent of which nutrient is limiting. For example, zooplankton abundance and export to higher trophic levels decreased in both N- and P-limited systems by a similar amount, because of the competition with viruses for bacteria. While we observed a difference in the increase of inorganic nutrients due to viruses between the N- and P-limited systems, it was not related to the differential response of nutrient release to viruses between these two systems. Understanding the extent of bottom-up (through nutrient release) and top-down (through mortality) control of the community by viruses thus seems a necessary step to explain the effect of nutrient limitation, both in models and empirical work.

We focused on lytic viruses, as temperate viruses do not contribute directly to the viral shunt. In our context lysogens could, for example, provide hosts with protection against secondary infection. This possibility would in our model be equivalent to reduced burst sizes or lytic rates, already considered in the wide range of parameters we used here. We focused, in particular, on the steady-state dynamics of the lytic system, and some of the equilibrium expressions provided clear evidence of bottom-up vs. top-down control. For example, bacterial abundance at equilibrium was only determined by parameters regulating viral lysis and decay, indicating that a top-down control was dominant. However, this was not necessarily true of transient dynamics. In moving forward, it will be important to understand how representative equilibrium values really are at the surface, which undergoes daily cycles in UV exposure and annual cycles in temperature that affect both viral decay and bacterial growth (Suttle and Chen, 1992; Apple et al., 2006). The information we lose by giving up on obtaining symbolic steady-state expressions may well be compensated for by the insights we gain by allowing for variation in nutrient content, or by including an infected class.

While adding pertinent features could help in understanding how bacteria, viruses, and nutrients interact in the ocean, our model proved sufficient to obtain realistic values for all variables. In particular, realistic carbon values emerged in some communities, from only two terms describing the production of sinking materials by predators of zooplankton, and through lysis. We found that carbon sink increased proportionally with viral abundance in log space, emphasizing the need to better understand the impact of viruses on global nutrient cycles and climate change (Danovaro et al., 2011). Although good estimates of global carbon sink rates—and thus carbon sequestration from the atmosphere—are important to understand the carbon cycle, carbon sink measurements are still difficult and rely on local sediment traps (Honjo et al., 2008). Depending on depth, the time interval between trap placement and retrieval can be long, limiting the number of surveyed locations. If the linear relationship between viruses and carbon sink unveiled here is confirmed by field studies, there could be an exciting opportunity to estimate carbon sink from virus-like particles count in water samples, regardless of the limiting nutrient. Models like ours could be used for this estimation by fine-tuning it to a specific survey location, in order to be able to detect, e.g., inter-annual variability, which is estimated at 50% (Gruber et al., 2002).

Finally, the VPRs and power-law coefficients that emerged from our models, consistent with empirical values (Wigington et al., 2016), suggest the potential of these models to accurately represent real-life dynamics rather than simply matching steady state and target values. The widespread coexistence and stability we observed, as well as realistic emergent properties, show that these models can be convenient and reliable tools to better understand the complex feedbacks present in marine microbial communities. The sub-linear relationship between bacteria and viruses we observed, which translated into a negative correlation between VPR and bacterial abundance, has been the subject of different interpretations (for example, as a sign of the increasing prevalence of lysogeny at high bacterial abundance Knowles et al., 2016). Our results show that lytic dynamics alone can be consistent with a sub-linear relationship between viruses and bacteria. Interestingly, we did not find a significant relationship between viral and bacterial abundance for heterotrophic bacteria in the N-limited model, emphasizing the importance of the limiting nutrient (in this case, organic N) in determining bacterium-virus relationships.

## Data Availability Statement

The datasets generated for this study are available on request to the corresponding author.

## Author Contributions

JP conceived the original idea. JP, CT, and JB designed research and wrote the manuscript. JP adapted the models, performed research, and contributed analytical tools.

### Conflict of Interest

The authors declare that the research was conducted in the absence of any commercial or financial relationships that could be construed as a potential conflict of interest.

## References

[B1] Ammerman J. W. Hood R. R. Case D. A. Cotner J. B. (2003). Phosphorus deficiency in the Atlantic: an emerging paradigm in oceanography. Eos Trans. Am. Geophys. Union 84:165 10.1029/2003EO180001

[B2] Antia A. N. Koeve W. Fischer G. Blanz T. Schulz-Bull D. Schölten J. (2001). Basin-wide particulate carbon flux in the Atlantic Ocean: regional export patterns and potential for atmospheric CO2 sequestration. Global Biogeochem. Cycles 15, 845–862. 10.1029/2000GB001376

[B3] Apple J. K. Del Giorgio P. A. Kemp W. M. (2006). Temperature regulation of bacterial production, respiration, and growth efficiency in a temperate salt-marsh estuary. Aquat. Microbial. Ecol. 43, 243–254. 10.3354/ame043243

[B4] Baar H. J. W. (1994). von Liebig 's law of the minimum and plankton ecology. Prog. Oceanogr. 33, 347–386. 10.1016/0079-6611(94)90022-1

[B5] Barange M. Butenschön M. Yool A. Beaumont N. Fernandes J. A. Martin A. P. (2017). The cost of reducing the North Atlantic Ocean biological carbon pump. Front. Mar. Sci. 3:290 10.3389/fmars.2016.00290

[B6] Bergh Ø. Børsheim K. Y. Bratbak G. Heldal M. (1989). High abundance of viruses found in aquatic environments. Nature. 340, 467–468. 10.1038/340467a0 2755508

[B7] Bonachela J. A. Klausmeier C. A. Edwards K. F. Litchman E. Levin S. A. (2015). The role of phytoplankton diversity in the emergent oceanic stoichiometry. J. Plankton Res. 38, 1021–1035. 10.1093/plankt/fbv087

[B8] Bongiorni L. Magagnini M. Armeni M. Noble R. Danovaro R. (2005). Viral production, decay rates, and life strategies along a trophic gradient in the north Adriatic sea. Appl. Environ. Microbiol. 71, 6644–6650. 10.1128/AEM.71.11.6644-6650.2005 16269692 PMC1287695

[B9] Brzezinski M. A. M. Dickson L. Nelson D. M. Sambrotto R. (2003). Ratios of Si, C and N uptake by microplankton in the Southern Ocean. Deep Sea Res. II Topic. Studies Oceanograp. 50, 619–633. 10.1016/S0967-0645(02)00587-8

[B10] Chibani-chennoufi S. Bruttin A. Brüssow H. Dillmann M. Bru H. (2004). Phage-host interaction : an ecological perspective. J. Bacteriol. 186, 3677–3686. 10.1128/JB.186.12.3677-3686.2004 15175280 PMC419959

[B11] Choua M. Bonachela J. A. (2019). Ecological and evolutionary consequences of viral plasticity. Am. Nat. 193, 346–358. 10.1086/701668 30794445

[B12] Danovaro R. Corinaldesi C. Dell'Anno A. Fuhrman J. A. Middelburg J. Noble R. T.. (2011). Marine viruses and global climate change. FEMS Microbiol. Rev. 35, 993–1034. 10.1111/j.1574-6976.2010.00258.x 21204862

[B13] Follows M. J. Dutkiewicz S. Grant S. Chisholm S. W. (2007). Emergent biogeography of microbial communities in a model ocean. Science 315, 1843–1846. 10.1126/science.1138544 17395828

[B14] Fuhrman J. (1992). Bacterioplankton roles in cycling of organic matter: the microbial food web, in Primary Productivity and Biogeochemical Cycles in the Sea, eds Falkowski P. G. Woodhead A. D. Vivirito K. (Boston, MA: Springer), 361–383.

[B15] Fuhrman J. A. (1999). Marine viruses and their biogeochemical and ecological effects. Nature 399, 541–548. 10.1038/21119 10376593

[B16] Gobler C. J. Hutchins D. A. Fisher N. S. Cosper E. M. Sañudo-Wilhelmy S. A. (1997). Release and bioavailability of C, N, P, Se, and Fe following viral lysis of a marine chrysophyte. Limnol. Oceanogr. 42, 1492–1504. 10.4319/lo.1997.42.7.1492

[B17] Graziano L. M. Geider R. J. Li W. K. W. Olaizola M. (1996). Nitrogen limitation of North Atlantic phytoplankton: analysis of physiological condition in nutrient enrichment experiments. Aquat. Microbial. Ecol. 11, 53–64. 10.3354/ame011053

[B18] Gruber N. Keeling C. D. Bates N. R. (2002). Interannual variability in the North Atlantic Ocean carbon sink. Science 298, 2374–2378. 10.1126/science.1077077 12493911

[B19] Hadas H. Einav M. Fishov I. Zaritsky A. (1997). Bacteriophage T4 development depends on the physiology of its host *Escherichia coli*. Microbiology 143, 179–185. 10.1099/00221287-143-1-179 9025292

[B20] Honjo S. H. Manganini S. J. Krishfield R. A. Francois R. (2008). Particulate organic carbon fluxes to the ocean interior and factors controlling the biological pump: a synthesis of global sediment trap programs since 1983. Prog. Oceanogr. 76, 217–285. 10.1016/j.pocean.2007.11.003

[B21] Johnson Z. I. Zinser E. R. Coe A. McNulty N. P. Woodward E. M. Chisholm S. W. (2006). Niche partitioning among prochlorococcus ecotypes along ocean-scale environmental gradients. Science 311, 1737–1740. 10.1126/science.1118052 16556835

[B22] Jover L. F. Effler T. C. Buchan A. Wilhelm S. W. Weitz J. S. (2014). The elemental composition of virus particles: implications for marine biogeochemical cycles. Nat. Rev. Microbiol. 12, 519–528. 10.1038/nrmicro3289 24931044

[B23] Keller D. Hood R. (2013). Comparative simulations of dissolved organic matter cycling in idealized oceanic, coastal, and estuarine surface waters. J. Mar. Syst. 109, 109–128. 10.1016/j.jmarsys.2012.01.002

[B24] Keller D. P. Hood R. R. (2011). Modeling the seasonal autochthonous sources of dissolved organic carbon and nitrogen in the upper Chesapeake Bay. Ecol. Modell. 222, 1139–1162. 10.1016/j.ecolmodel.2010.12.014

[B25] Klausmeier C. A. Litchman E. Daufresne T. Levin S. A. (2004). Optimal nitrogen-to-phosphorus stoichiometry of phytoplankton. Nature 429, 171–174. 10.1038/nature02454 15141209

[B26] Knowles B. Silveira C. B. Bailey B. A. Barott K. Cantu V. A. Cobián-Güemes A. G.. (2016). Lytic to temperate switching of viral communities. Nature 531, 466–470. 10.1038/nature17193 26982729

[B27] Lee B.-G. Fisher N. S. (1994). Effects of sinking and zooplankton grazing on the release of elements from planktonic debris. Mar. Ecol. Prog. Ser. 110, 271–281. 10.3354/meps110271

[B28] Letscher R. T. Hansell D. A. Carlson C. A. Lumpkin R. Knapp A. N. (2013). Dissolved organic nitrogen in the global surface ocean: distribution and fate. Global Biogeochem. Cycles 27, 141–153. 10.1029/2012GB004449

[B29] Leynaert A. Tréguer P. Lancelot C. Rodier M. (2001). Silicon limitation of biogenic silica production in the Equatorial Pacific. Deep Sea Res. I Oceanograph. Res. Papers 48, 639–660. 10.1016/S0967-0637(00)00044-3

[B30] Li W. K. W. (1998). Annual average abundance of heterotrophic bacteria and *Synechococcus* in surface ocean waters. Limnol. Oceanogr. 43, 1746–1753. 10.4319/lo.1998.43.7.1746

[B31] Litchman E. Klausmeier C. A. Schofield O. M. Falkowski P. G. (2007). The role of functional traits and trade-offs in structuring phytoplankton communities: scaling from cellular to ecosystem level. Ecol. Lett. 10, 1170–1181. 10.1111/j.1461-0248.2007.01117.x 17927770

[B32] Maat D. S. Brussaard C. P. D. (2016). Both phosphorus-and nitrogen limitation constrain viral proliferation in marine phytoplankton. Aquat. Microbial. Ecol. 77, 87–97. 10.3354/ame01791

[B33] Marsay C. M. Sanders R. J. Henson S. A. Pabortsava K. Achterberg E. P. Lampitt R. S. (2015). Attenuation of sinking particulate organic carbon flux through the mesopelagic ocean. Proc. Natl. Acad. Sci. U.S.A. 112, 1089–1094. 10.1073/pnas.1415311112 25561526 PMC4313834

[B34] Mather R. L. Reynolds S. E. Wolff G. A. Williams R. G. Torres-Valdés S. Woodward E. M. S. (2008). Phosphorus cycling in the North and South Atlantic Ocean subtropical gyres. Nat. Geosci. 1, 439–443. 10.1038/ngeo232

[B35] McKay M. D. Beckman R. J. Conover W. J. (1979). Comparison of three methods for selecting values of input variables in the analysis of output from a computer code. Technometrics 21, 239–245. 10.1080/00401706.1979.10489755

[B36] Middelboe M. Jørgensen N. O. G. Kroer N. (1996). Effects of viruses on nutrient turnover and growth efficiency of noninfected marine bacterioplankton. Appl. Environ. Microbiol. 62, 1991–1997. 10.1128/AEM.62.6.1991-1997.1996 16535334 PMC1388872

[B37] Middelboe M. Lyck P. G. (2002). Regeneration of dissolved organic matter by viral lysis in marine microbial communities. Aquat. Microbial. Ecol. 27, 187–194. 10.3354/ame027187

[B38] Miki T. Nakazawa T. Yokokawa T. Nagata T. (2008). Functional consequences of viral impacts on bacterial communities: a food-web model analysis. Freshw. Biol. 53, 1142–1153. 10.1111/j.1365-2427.2007.01934.x

[B39] Monod J. (1950). La technique de culture continue: théorie et applications. Annls Inst. Pasteur. 79, 390–410.

[B40] Moore J. K. Doney S. C. Glover D. M. Fung I. (2002). Iron cycling and nutrient-limitation patterns in surface waters of the world ocean. Deep-Sea Res. II Topic. Studies Oceanograp. 49, 463–507. 10.1016/S0967-0645(01)00109-6

[B41] Morel F. M. M. (1987). Kinetics of nutrient uptake and growth in phytoplankton. J. Phycol. 23, 137–150. 10.1111/j.1529-8817.1987.tb04436.x

[B42] Mouw C. B. Barnett A. McKinley G. A. Gloege L. Pilcher D. (2016). Global ocean particulate organic carbon flux merged with satellite parameters. Earth Syst. Sci. Data 8, 531–541. 10.5194/essd-8-531-2016

[B43] Parada P. Herndl G. J. Weinbauer M. (2006). Viral burst size of heterotrophic prokaryotes in aquatic systems. J. Marine Biol. Assoc. U.K. 86, 613–621. 10.1017/S002531540601352X

[B44] Parikka K. J. Le Romancer M. Wauters N. Jacquet S. (2017). Deciphering the virus-to-prokaryote ratio (VPR): insights into virus–host relationships in a variety of ecosystems. Biol. Rev. 92, 1081–1100. 10.1111/brv.12271 27113012

[B45] Puxty R. J. Millard A. D. Evans D. J. Scanlan D. J. (2016). Viruses inhibit CO 2 fixation in the most abundant phototrophs on earth. Curr. Biol. 26, 1585–1589. 10.1016/j.cub.2016.04.036 27291056

[B46] Schartau M. Landry M. R. Armstrong R. A. (2010). Density estimation of plankton size spectra: a reanalysis of IronEx II data. J. Plankton Res. 32, 1167–1184. 10.1093/plankt/fbq072

[B47] Shelford E. J. Middelboe M. Møller E. F. Suttle C. A. (2012). Virus-driven nitrogen cycling enhances phytoplankton growth. Aquat. Microbial. Ecol. 66, 41–46. 10.3354/ame01553

[B48] Small L. F. Fowler S. W. Moore S. A. LaRosa J. (1983). Dissolved and fecal pellet carbon and nitrogen release by zooplankton in tropical waters. Deep Sea Res Oceanograph. Res. Papers 30, 1199–1220. 10.1016/0198-0149(83)90080-8

[B49] Sterner R. W. Elser J. J. (2002). Ecological Stoichiometry: The Biology of Elements from Molecules to the Biosphere. Princeton, NJ: Princeton University Press 10.1515/9781400885695

[B50] Suttle C. A. (2007). Marine viruses — major players in the global ecosystem. Nat. Rev. Microbiol. 5, 801–812. 10.1038/nrmicro1750 17853907

[B51] Suttle C. A. Chan A. M. Cottrell M. T. (1990). Infection of phytoplankton by viruses and reduction of primary productivity. Nature 347, 467–469. 10.1038/347467a0

[B52] Suttle C. A. Chen F. (1992). Mechanisms and rates of decay of marine viruses in seawater. Appl. Environ. Microbiol. 58, 3721–3729. 10.1128/AEM.58.11.3721-3729.1992 16348812 PMC183166

[B53] Thingstad T. F. (2000). Elements of a theory for the mechanisms controlling abundance, diversity, and biogeochemical role of lytic bacterial viruses in aquatic systems. Limnol. Oceanogr. 45, 1320–1328. 10.4319/lo.2000.45.6.1320

[B54] Weinbauer M. Bonilla-Findji O. Chan A. M. Dolan J. R. Short S. M. Šimek K. (2011). *Synechococcus* growth in the ocean may depend on the lysis of heterotrophic bacteria. J. Plankton Res. 33, 1465–1476. 10.1093/plankt/fbr041

[B55] Weitz J. (2015). Quantitative Viral Ecology: Dynamics of Viruses and Their Microbial Hosts. Princeton, NJ: Princeton University Press.

[B56] Weitz J. S. Stock C. A. Wilhelm S. W. Bourouiba L. Coleman M. L. Buchan A.. (2015). A multitrophic model to quantify the effects of marine viruses on microbial food webs and ecosystem processes. ISME J. 9, 1352–1364. 10.1038/ismej.2014.220 25635642 PMC4438322

[B57] Weitz J. S. Wilhelm S. W. (2012). Ocean viruses and their effects on microbial communities and biogeochemical cycles. F1000 Biol. Rep. 4:17. 10.3410/B4-17 22991582 PMC3434959

[B58] Wigington C. H. Sonderegger D. Brussaard C. P. D. Buchan A. Finke J. F. Fuhrman J. A.. (2016). Re-examination of the relationship between marine virus and microbial cell abundances. Nat. Microbiol. 1:15024. 10.1038/nmicrobiol.2015.24 27572161

[B59] Wilhelm W. Suttle C. A. (1999). Viruses and nutrient cycles in the Sea. Bioscience 49, 781–788. 10.2307/1313569

[B60] Yool A. Popova E. E. Anderson T. R. (2013). MEDUSA-2.0: an intermediate complexity biogeochemical model of the marine carbon cycle for climate change and ocean acidification studies. Geosci. Model Dev. 6, 1767–1811. 10.5194/gmd-6-1767-2013

[B61] You L. Suthers P. F. Yin J. (2002). Effects of *Escherichia coli* physiology on growth of phage T7 *in vivo* and *in silico*. J. Bacteriol. 184, 1888–1894. 10.1128/JB.184.7.1888-1894.2002 11889095 PMC134924

[B62] Zeng Q. Chisholm S. W. (2012). Marine viruses exploit their host's two-component regulatory system in response to resource limitation. Curr. Biol. 22, 124–128. 10.1016/j.cub.2011.11.055 22244998

