# Supplementary material for: Impact of Lytic Phages on Phosphorus- vs. Nitrogen-Limited Marine Microbes
Source: Front Microbiol. 2020 Feb 21;11:221. doi: 10.3389/fmicb.2020.00221 (PMC7047511; doi:10.3389/fmicb.2020.00221)
Supplement: Supplementary file 1 [file Data_Sheet_1.pdf]

Impact of lytic phages on phosphorus- versus nitrogen-limited marine microbes

Julie Pourtois[1,2], Corina E. Tarnita[1], and Juan A. Bonachela[3,*]

[1]Department of Ecology and Evolutionary Biology, Princeton University, Princeton, NJ 08544, USA

[2]Department of Biology, Stanford University, Stanford, CA 94305, USA

[3]Department of Ecology, Evolution, and Natural Resources, Rutgers University, New Brunswick, NJ 08901, USA

* To whom correspondence should be addressed: juan.bonachela@rutgers.edu

Appendix A

Nitrogen-limited system

$$\frac{dH}{dt} = \overbrace{\frac{r_H H N_{org}}{K_{org} + N_{org}}}^{N_{org}\, uptake} - \overbrace{\phi_H H V_H}^{lysis} - \overbrace{\gamma_H H Z}^{grazing} - \overbrace{\lambda_H H}^{respiration} - \overbrace{\sigma_H H}^{organic\ loss} \tag{A1}$$

$$\frac{dC}{dt} = \overbrace{\frac{r_C C N_{in}}{K_{in} + N_{in}}}^{N_{in} uptake} - \overbrace{\phi_C C V_C}^{lysis} - \overbrace{\gamma_C C Z}^{grazing} - \overbrace{\lambda_C C}^{respiration} - \overbrace{\sigma_C C}^{organic\ loss} \tag{A2}$$

$$\frac{dZ}{dt} = \overbrace{\frac{p_g Z(b_H \gamma_H H + b_C \gamma_C C)}{b_Z}}^{grazing} - \overbrace{\lambda_Z Z}^{respiration} - \overbrace{\gamma_Z Z^2}^{predation} \tag{A3}$$

$$\frac{dV_H}{dt} = \overbrace{\beta_H \phi_H H V_H}^{lysis} - \overbrace{\theta_H V_H}^{decay} \tag{A4}$$

$$\frac{dV_C}{dt} = \overbrace{\beta_C \phi_C C V_C}^{lysis} - \overbrace{\theta_C V_C}^{decay} \tag{A5}$$

$$\frac{dN_{in}}{dt} = \overbrace{-\omega(N_{in} - N_{sub})}^{exchange} + \overbrace{\frac{b_H(1-\epsilon_H) r_H H N_{org}}{\epsilon_H (K_{org} + N_{org})}}^{H\ uptake} - \overbrace{\frac{b_C r_C C N_{in}}{K_{in} + N_{in}}}^{C\ uptake} + \overbrace{b_Z \lambda_Z Z}^{Z\ respiration}$$

$$+ \overbrace{b_H \lambda_H H}^{H\ respiration} + \overbrace{b_C \lambda_C C}^{H\ respiration} + \overbrace{Z\big(1 - p_g - p_{ex}\big)(b_H \gamma_H H + b_C \gamma_C C)}^{grazing} \tag{A6}$$

$$\frac{dN_{org}}{dt} = \overbrace{-\frac{b_H r_H H N_{org}}{\epsilon_H (K_{org} + N_{org})}}^{H\ uptake} + \overbrace{b_V \theta_H V_H}^{V_H\ decay} + \overbrace{b_V \theta_C V_C}^{V_C\ decay} + \overbrace{(b_H - b_V \beta_H)\phi_H H V_H}^{H\ lysis}$$

$$+ \overbrace{(b_C - b_V \beta_C)\phi_C C V_C}^{C\ lysis} + \overbrace{b_H \sigma_H H}^{H\ organic\ loss} + \overbrace{b_C \sigma_C C}^{C\ organic\ loss}$$

$$+ \overbrace{p_{ex} Z (b_H \gamma_H H + b_C \gamma_C C)}^{grazing} \tag{A7}$$

*Phosphorus-limited syste*m

$$\frac{dH}{dt} = \overbrace{\frac{r_H H P_{in}}{K_{H,in} + P_{in}}}^{P_{in}\ uptake} - \overbrace{\phi_H H V_H}^{lysis} - \overbrace{\gamma_H H Z}^{grazing} - \overbrace{\lambda_H H}^{respiration} - \overbrace{\sigma_H H}^{organic\ loss} \tag{A8}$$

$$\frac{dC}{dt} = \overbrace{\frac{r_C C P_{in}}{K_{C,in} + P_{in}}}^{P_{in}\ uptake} - \overbrace{\phi_C C V_C}^{lysis} - \overbrace{\gamma_C C Z}^{grazing} - \overbrace{\lambda_C C}^{respiration} - \overbrace{\sigma_C C}^{organic\ loss} \tag{A9}$$

$$\frac{dZ}{dt} = \frac{\overbrace{p_g Z (q_H \gamma_H H + q_C \gamma_C C)}^{grazing}}{q_Z} - \overbrace{\lambda_Z Z}^{respiration} - \overbrace{\gamma_Z Z^2}^{predation} \tag{A10}$$

$$\frac{dV_H}{dt} = \overbrace{\beta_H \phi_H H V_H}^{lysis} - \overbrace{\theta_H V_H}^{decay} \tag{A11}$$

$$\frac{dV_C}{dt} = \overbrace{\beta_C \phi_C C V_C}^{lysis} - \overbrace{\theta_C V_C}^{decay} \quad \text{(A12)}$$

$$\frac{dP_{in}}{dt} = -\overbrace{\omega(P_{in} - P_{sub})}^{exchange} - \overbrace{\frac{q_H r_H H P_{in}}{K_{H,in} + P_{in}}}^{H\ uptake} - \overbrace{\frac{q_C r_C C P_{in}}{K_{C,in} + P_{in}}}^{C\ uptake} + \overbrace{q_Z \lambda_Z Z}^{Z\ respiration} + \overbrace{q_H \lambda_H H}^{H\ respiration}$$
$$+ \overbrace{q_C \lambda_C C}^{C\ respiration} + \overbrace{(1 - p_g - p_{ex})(q_H \gamma_H H Z + q_C \gamma_C C Z)}^{grazing} + \overbrace{\psi_H H P_{org}}^{remineralization}$$
$$+ \overbrace{\psi_C C P_{org}}^{remineralization} \quad \text{(A13)}$$

$$\frac{dP_{org}}{dt} = \overbrace{q_V \theta_H V_H}^{V_H\ decay} + \overbrace{q_V \theta_C V_C}^{V_C\ decay} + \overbrace{(q_H - q_V \beta_H)\phi_H H V_H}^{H\ lysis} + \overbrace{(q_C - q_V \beta_C)\phi_C C V_C}^{C\ lysis}$$
$$+ \overbrace{q_H \sigma_H H}^{H\ organic\ loss} + \overbrace{q_C \sigma_C C}^{C\ organic\ loss} + \overbrace{p_{ex} Z(q_H \gamma_H H + q_C \gamma_C C)}^{grazing}$$
$$- \overbrace{\psi_H H P_{org}}^{remineralization} - \overbrace{\psi_C C P_{org}}^{remineralization} \quad \text{(A14)}$$

Fluxes

The equations below provide expressions for productivity (P), nutrient release (NR), nutrient export to higher trophic levels (NE), and carbon sink (CS), for the N-limited systems. The equations for the P-limited systems are similar, and just require replacing the N-specific parameters with their phosphorus equivalent (e.g. $b_H \leftrightarrow q_H$, $b_{C:N} \leftrightarrow b_{C:P}$, etc).

$$P = \frac{b_H r_H H N_{org}}{K_{org} + N_{org}} + \frac{b_C r_C C N_{in}}{K_{in} + N_{in}} \quad \text{(A15)}$$

$$NR = (b_H - b_V \beta_H)\phi_H H V_H + (b_C - b_V \beta_C)\phi_C C V_C + p_{ex} Z(b_H \gamma_H H + b_C \gamma_C C) \quad \text{(A16)}$$

$$NE = b_Z \gamma_Z Z^2 \quad \text{(A17)}$$

$$CS = b_Z \gamma_Z Z^2 p_f z_{C:N} + (b_H b_{C:N} - b_V v_{C:N} \beta_H)\phi_H H V_H p_{rec}$$
$$+ (b_C b_{C:N} - b_V v_{C:N} \beta_C)\phi_C C V_C p_{rec} \quad \text{(A18)}$$

Appendix B

Equilibrium expressions

N-limited, with viruses

$$H = \frac{\theta_H}{\beta_H \phi_H} \tag{B1}$$

$$C = \frac{\theta_C}{\beta_C \phi_C} \tag{B2}$$

$$Z = \frac{p_g(b_H \gamma_H H + b_C \gamma_C C)}{b_Z \gamma_Z} - \frac{\lambda_Z}{\gamma_Z} \tag{B3}$$

$$N_{in} = N_{sub} - \frac{b_Z \gamma_Z Z^2}{\omega} \tag{B4}$$

$$V_C = \frac{r_C N_{in}}{\phi_C (N_{in} + K_{in})} - \frac{\lambda_C}{\phi_C} - \frac{\sigma_C}{\phi_C} - \frac{\gamma_C Z}{\phi_C} \tag{B5}$$

$$V_H = \frac{x}{y} \tag{B6}$$

where:

$$x = -\frac{b_H H(\gamma_H Z + \lambda_H + \sigma_H)}{\epsilon_H} + b_V \theta_C V_C + (b_C - b_V \beta_C)\phi_{VC} C V_C + p_{ex} b_H \gamma_H H Z + p_{ex} b_C \gamma_C C Z + b_H \sigma_H H + b_C \sigma_C C \tag{B7}$$

$$y = \frac{b_H \phi_{V_H} H}{\epsilon_H} - b_V \theta_H - (b_H - b_V \beta_H)\phi_{V_H} H \tag{B8}$$

$$N_{org} = \frac{K_{org}(\phi_{V_H} V_H + \gamma_H Z + \lambda_H + \sigma_H)}{r_H - \phi_{V_H} V_H - \gamma_H Z - \lambda_H - \sigma_H} \tag{B9}$$

N-limited, without viruses

We find the equilibrium expression for Z by solving a polynomial of the third degree of the form $C_3Z^3 + C_2Z^2 + C_1Z + C_0 = 0$ where:

$$C_3 = \frac{\gamma_C b_Z \gamma_Z}{\omega} \quad \text{(B10)}$$

$$C_2 = -\frac{b_Z \gamma_Z (r_C - \lambda_C - \sigma_C)}{\omega} \quad \text{(B11)}$$

$$C_1 = -K_{in}\gamma_C - N_{sub}\gamma_C \quad \text{(B12)}$$

$$C_0 = N_{sub}(r_C - \lambda_C - \sigma_C) - K_{in}(\lambda_C + \sigma_C) \quad \text{(B13)}$$

We solved for Z numerically. We then found the equilibrium expressions of the remaining variables.

$$N_{in} = N_{sub} - \frac{b_Z \gamma_Z Z^2}{\omega} \quad \text{(B14)}$$

$$N_{org} = \frac{K_{org}(\gamma_H Z + \lambda_H + \sigma_H)}{r_H - \gamma_H Z - \lambda_H - \sigma_H} \quad \text{(B15)}$$

$$H = \frac{\sigma_C b_Z (\lambda_Z + \gamma_Z Z) + p_{ex} b_Z Z \gamma_C (\lambda_Z + \gamma_Z Z)}{p_G \gamma_C \left(\frac{b_H r_H N_{org}}{\epsilon_H (K_{org} + N_{org})} - b_H \sigma_H + \frac{\sigma_C b_H \gamma_H}{\gamma_C} \right)} \quad \text{(B16)}$$

$$C = \frac{b_Z(\lambda_Z + \gamma_Z Z)}{p_G b_C \gamma_C} - \frac{b_H \gamma_H H}{b_C \gamma_C} \quad \text{(B17)}$$

P-limited, with viruses

$$H = \frac{\theta_H}{\beta_H \phi_H} \quad \text{(B18)}$$

$$C = \frac{\theta_C}{\beta_C \phi_C} \tag{B19}$$

$$Z = \frac{p_g(q_H\gamma_H H + q_C\gamma_C C)}{q_Z\gamma_Z} - \frac{\lambda_Z}{\gamma_Z} \tag{B20}$$

$$P_{in} = P_{sub} - \frac{q_Z\gamma_Z Z^2}{\omega} \tag{B21}$$

$$V_H = \frac{r_H P_{in}}{\phi_H(P_{in} + K_{H,in})} - \frac{\lambda_H}{\phi_H} - \frac{\sigma_H}{\phi_H} - \frac{\gamma_H Z}{\phi_H} \tag{B22}$$

$$V_C = \frac{r_C P_{in}}{\phi_C(P_{in} + K_{C,in})} - \frac{\lambda_C}{\phi_C} - \frac{\sigma_C}{\phi_C} - \frac{\gamma_C Z}{\phi_C} \tag{B23}$$

$$P_{org} = \frac{q_H\sigma_H H + q_C\sigma_C C + p_{ex}Z(\gamma_H q_H H + \gamma_C q_C C) + \theta_H V_H q_V + \theta_C V_C q_V}{\psi_H H + \psi_C C} + \frac{(q_H - \beta_H q_V)\phi_H V_H H + (q_C - \beta_C q_V)\phi_C V_C C}{\psi_H H + \psi_C C} \tag{B24}$$

P-limited, without viruses

We find the equilibrium expression for Z by solving a polynomial of the third degree of the form $C_3Z^3 + C_2Z^2 + C_1Z + C_0 = 0$ where:

$$\xi = q_Z\gamma_Z\omega \tag{B25}$$

$$C_3 = -\gamma_H\xi \tag{B26}$$

$$C_2 = \xi(r_H - \lambda_H - \sigma_H) \tag{B27}$$

$$C_1 = \gamma_H(P_{sub} + K_{H,in}) \tag{B28}$$

$$C_0 = \lambda_H P_{sub} + \lambda_H K_{H,in} + \sigma_H P_{sub} + \sigma_H K_{H,in} - r_H P_{sub} \tag{B29}$$

We solved for Z numerically. We then found the equilibrium expressions of the remaining variables.

$$P_{org} = \frac{q_H \sigma_H}{\psi_H} + \frac{p_{ex} \gamma_H q_H Z}{\psi_H} \quad \text{(B30)}$$

$$H = \frac{q_Z(\lambda_Z + \gamma_Z Z)}{p_g q_H \gamma_H} \quad \text{(B31)}$$

$$P_{in} = P_{sub} - \frac{q_Z \gamma_Z Z^2}{\omega} = P_{sub} - \xi Z^2 \quad \text{(B32)}$$

Appendix C

Table C-1. Parameter ranges.

Process	Symbol	Meaning	Minimum	Maximum	Units
H nutrient uptake	r_H	Maximum growth rate	0.5	5	day^{-1}
	q_H	H phosphorus content	10^{-11}	5×10^{-10}	μmol P/cell
	b_H	H nitrogen content	5×10^{-10}	4×10^{-9}	μmol N/cell
	$K_{H,in}$	P_{in} Half-saturation constant	0.005	1.5	μmol P
	K_{org}	N_{org} Half-saturation constant	0.25	1	μmol N/L
	ϵ_H	Efficiency	0.05	0.2	NA
	ψ_H	Phosphatase rate	2×10^{-13}	2×10^{-10}	$(cell \times day)^{-1}$
C nutrient uptake	r_C	Maximum growth rate	0.1	1.5	day^{-1}
	q_C	C phosphorus content	3×10^{-11}	2×10^{-10}	μmol P/cell
	b_C	C nitrogen content	5×10^{-10}	4×10^{-9}	μmol N/cell
	$K_{C,in}$	P_{in} Half-saturation constant	0.005	1.5	μmol P
	K_{in}	N_{in} Half-saturation constant	0.05	1	μmol N/L
	ψ_C	Phosphatase rate	2×10^{-13}	2×10^{-10}	$(cell \times day)^{-1}$
Respiration	λ_H	H respiration rate	0.005	0.05	day^{-1}
	λ_C	C respiration rate	0.0005	0.005	day^{-1}
	λ_Z	Z respiration rate	0.0005	0.01	day^{-1}
Cellular loss	σ_H	H exudation rate	0.0015	0.003	day^{-1}
	σ_C	C exudation rate	0.0005	0.0015	day^{-1}
Zooplankton	γ_H	H grazing rate	10^{-6}	10^{-4}	L/(cell × day)
	γ_C	C grazing rate	10^{-6}	10^{-4}	L/(cell × day)
	p_g	Growth efficiency	0.2	0.4	NA
	p_{ex}	Fraction excreted	0.2	0.4	NA
	q_z	Z phosphorus content	10^{-6}	2×10^{-5}	μmol P/zoopl.
	b_z	Z nitrogen content	5×10^{-5}	4×10^{-4}	μmol N/zoopl.
Viral lysis	ϕ_H	H lysis rate	10^{-13}	10^{-10}	L/(virus × day)
	ϕ_C	C lysis rate	10^{-13}	10^{-10}	L/(virus × day)
	β_H	H burst size	15	50	NA
	β_C	C burst size	20	150	NA
	q_v	V phosphorus content	0.5×10^{-13}	2×10^{-13}	μmol P/virus
	b_v	V nitrogen content	0.5×10^{-12}	3×10^{-12}	μmol N/virus
Viral decay	θ_H	V_H decay rate	0.05	1	day^{-1}
	θ_C	V_C decay rate	0.05	1	day^{-1}
Nutrient import and export	γ_Z	Consumption of Z (P)	10^{-6}	10^{-3}	L/(zoopl. × day)
	γ_Z	Consumption of Z (N)	10^{-8}	10^{-4}	L/(zoopl. × day)
	p_f	Fraction sink	0.05	0.3	NA
	$z_{C:N}$	C:N zooplankton ratio	4	8	NA
	$z_{C:P}$	C:P zooplankton ratio	60	200	NA
	$b_{C:N}$	C:N bacteria ratio	4	7	NA
	$b_{C:P}$	C:P bacteria ratio	64	112	NA
	$v_{C:N}$	C:N virus ratio	3	3.5	NA

$v_{C:P}$	C:P virus ratio	18	45	NA
p_{rec}	Fraction DOC refractory	0.1	0.3	NA
ω	Mixing rate	0.005	0.02	day^{-1}
P_{sub}	P_{in} in sublevel waters	0.05	0.2	µmol P/L
N_{sub}	N_{in} in sublevel waters	2.5	10	µmol N/L
N	Total phosphorus pool	0.08	0.18	µmol P/L

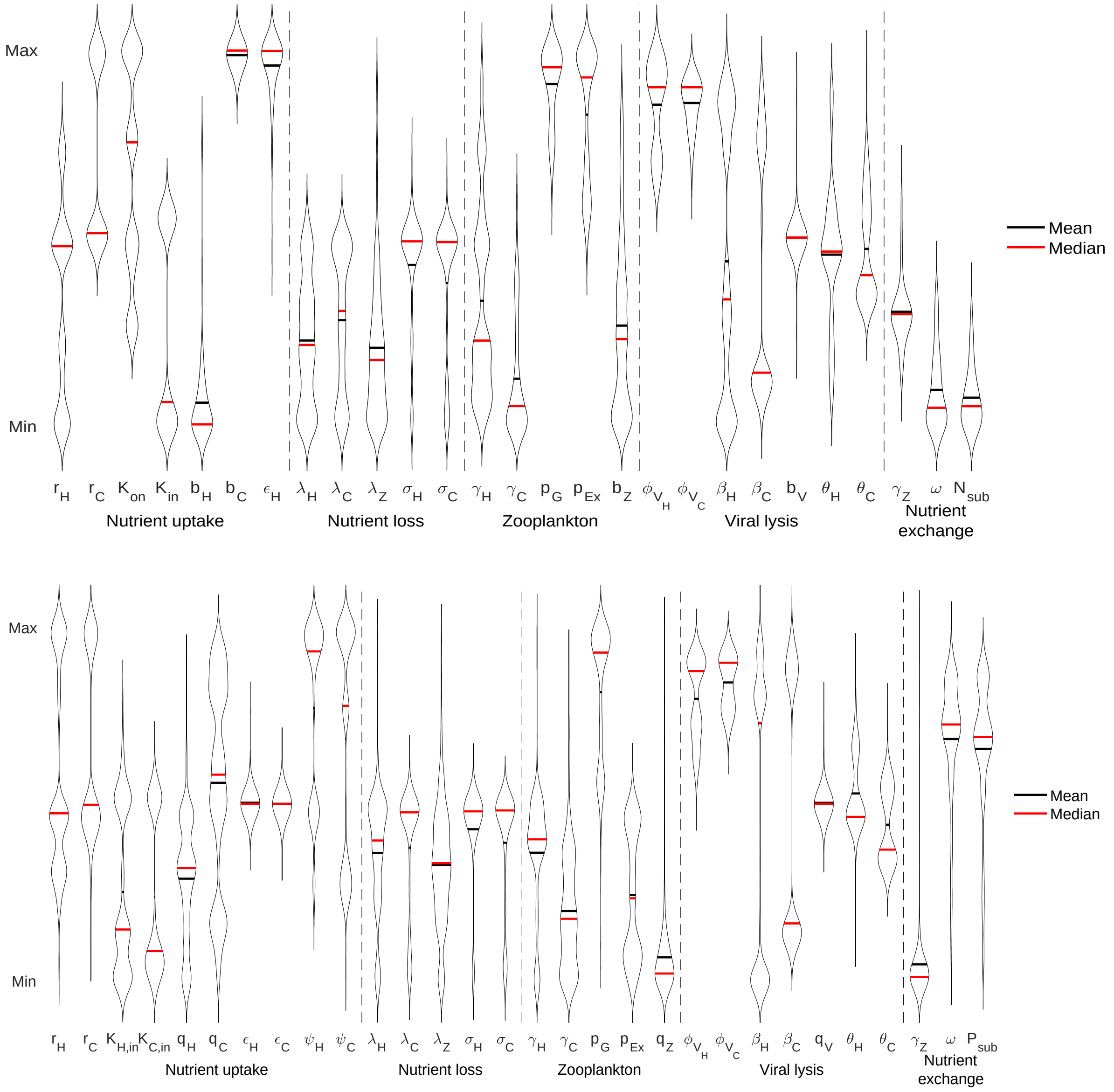

Figure C-1. Distribution of optimized parameters in log-space. Top: Nitrogen-limited system. Bottom: Phosphorus-limited system. Parameters in log-space were standardized by substracting the minimum of the range and then dividing by the range. Parameters are arranged according to the processes they relate to.

Appendix D

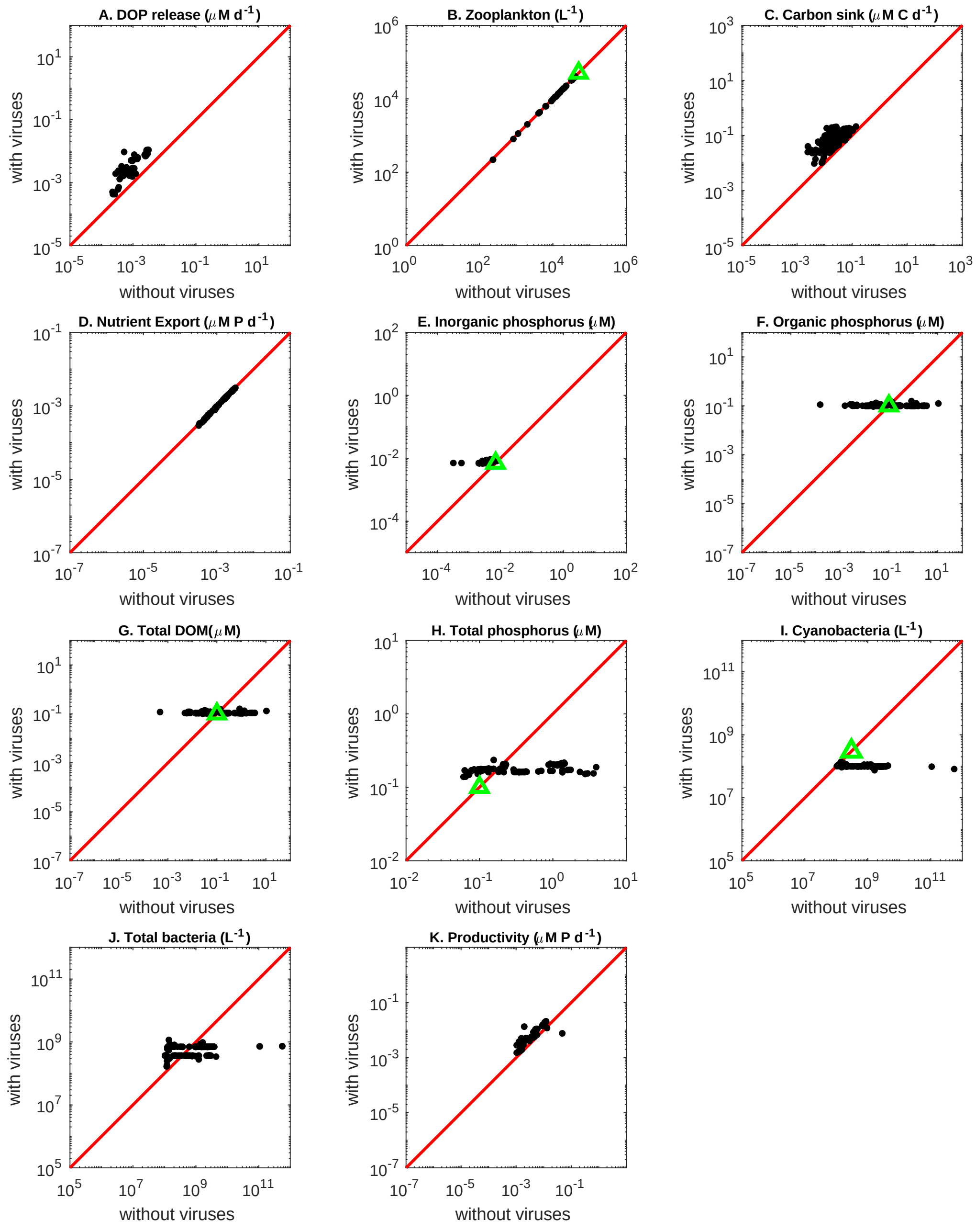

Figure D1. Effect of viruses on steady-state concentrations and fluxes for the phosphorus-limited system when heterotrophic bacteria are not represented in the virus-free system, but only cyanobacteria. The red line denotes the 1:1 line and the green triangles show target densities used in the optimization procedure. Each point stands for the steady concentration for one optimized parameter set. Points above and below the red line represent steady-state values that increased and decreased after introducing viruses respectively.
